# Composition-driven Cu-speciation and reducibility in Cu-CHA zeolite catalysts: a multivariate XAS/FTIR approach to complexity†
†Electronic supplementary information (ESI) available: Sample description and synthesis details, experimental setup for *in situ* XAS and FTIR spectroscopy, details on the MCR-ALS method, details on DFT-assisted XANES simulations, details on the determination of *N*_pure_ by PCA, MCR-ALS results for downsized and upsized component spaces, additional information to support the assignment of theoretical XANES curves, details on EXAFS analysis, details on IR spectral deconvolution. See DOI: 10.1039/c7sc02266b



**DOI:** 10.1039/c7sc02266b

**Published:** 2017-07-24

**Authors:** A. Martini, E. Borfecchia, K. A. Lomachenko, I. A. Pankin, C. Negri, G. Berlier, P. Beato, H. Falsig, S. Bordiga, C. Lamberti

**Affiliations:** a Department of Chemistry , NIS Centre and INSTM Reference Center , University of Turin , Via P. Giuria 7 , Turin , 10125 Italy . Email: elisa.borfecchia@unito.it; b Haldor Topsøe A/S , Haldor Topsøes Allé 1 , Kgs. Lyngby , 2800 Denmark; c European Synchrotron Radiation Facility , 71 avenue des Martyrs, CS 40220 , Grenoble Cedex 9 , 38043 France; d IRC “Smart Materials” , Southern Federal University , Zorge str. 5 , Rostov-on-Don , 344090 Russia; e Department of Chemistry , CrisDi Centre and INSTM Reference Center , University of Turin , Via P. Giuria 7 , Turin , 10125 Italy

## Abstract

The small pore Cu-CHA zeolite is attracting increasing attention as a versatile platform to design novel single-site catalysts for deNO_*x*_ applications and for the direct conversion of methane to methanol. Understanding at the atomic scale how the catalyst composition influences the Cu-species formed during thermal activation is a key step to unveil the relevant composition–activity relationships. Herein, we explore by *in situ* XAS the impact of Cu-CHA catalyst composition on temperature-dependent Cu-speciation and reducibility. Advanced multivariate analysis of *in situ* XANES in combination with DFT-assisted simulation of XANES spectra and multi-component EXAFS fits as well as *in situ* FTIR spectroscopy of adsorbed N_2_ allow us to obtain unprecedented quantitative structural information on the complex dynamics during the speciation of Cu-sites inside the framework of the CHA zeolite.

## Abstract 2

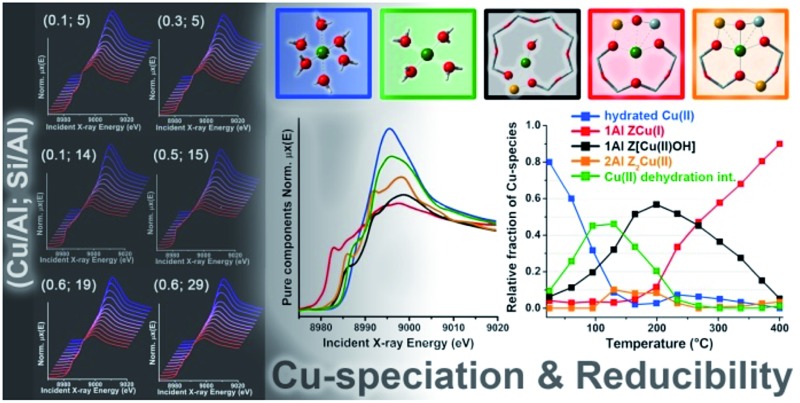
Multivariate XAS analysis and *in situ* FTIR enable an unprecedented quantitative understanding of the composition impact on temperature-dependent Cu-speciation and reducibility in Cu-CHA zeolite catalysts.

## 1 Introduction

Cu-exchanged chabazite (Cu-CHA) is currently the object of intensive research efforts to rationalize its outstanding performance in the NH_3_-assisted selective catalytic reduction (SCR) of NO_*x*_ (ref. 1–3) and its recently proven low-temperature activity in the direct conversion of methane to methanol.^4–9^ Among the multitude of zeolite topologies, CHA sticks out due to its structural simplicity. The framework is built up by an arrangement of double 6-membered rings (d6r), connected *via* 4-membered ring units, giving rise to the so-called CHA cages, which are accessible through 8-membered rings (8r). All of the tetrahedral sites (T-sites) occupied by Si or Al atoms are in crystallographically equivalent positions.^10,11^ Such a structural simplicity fostered the investigation of Cu-CHA as a model system to resolve fundamental questions of structure–performance relationships in metal-exchanged zeolite catalysis.^12^

Initial structural studies proposed that isolated Cu(i) or Cu(ii) cations exclusively occupy one extra-framework site in the plane of the 6-membered rings (6r).^13–15^ However, with the exponentially increasing number of studies in recent years, it has become evident that the CHA topology offers multiple docking sites for cations, and that the chemical properties (oxidation state, coordination geometry, and nuclearity) and location of Cu-species in Cu-CHA are strongly influenced by the sample composition in terms of the Si/Al ratio and Cu-loading.^1,16–18^ Importantly, Cu-speciation in the catalyst appears to be a dynamic process and largely driven by environmental conditions, such as temperature and gas feed composition. In the particular case of the presence of H_2_O or NH_3_, framework-interacting Cu species can easily convert into mobile complexes,^19–22^ freely suspended in the zeolite cages while undergoing redox chemistry, as proposed in a recently published low-temperature SCR mechanism.^23^ Advanced *in situ*/*operando* characterization approaches and appropriate analytic tools allowing one to manage complex spectroscopic datasets are thus mandatory to properly address the fickle nature of the active metals sites as well as to catch the dynamic host–guest interactions in these fascinating materials.

We have previously combined *in situ*/*operando* X-ray absorption (XAS) and emission spectroscopy (XES) to shed light on the structural and electronic properties of Cu-species formed during thermal activation,^24^ and during interaction with a complete SCR-feed^22^ or individual SCR-feed components.^3,19,25^ To limit the number of experimental variables, most of these studies were systematically conducted on a Cu-CHA catalyst with a fixed composition (Cu/Al ∼ 0.44; Si/Al ∼ 13), providing optimal SCR performance. However, as mentioned above, Cu-speciation in CHA is strongly affected by the catalyst composition (Cu/Al and Si/Al ratios), and in order to derive structure–activity relationships in catalytic processes, it is absolutely necessary to perform a systematic study on different catalyst compositions.

Recent studies^18,21,26,27^ indicate the presence of two major Cu-sites in activated Cu-CHA, with markedly different redox properties and relative abundance depending on the composition. These include reduction-resistant Z_2_Cu(ii) species hosted in the 6r of the CHA framework (where Z_2_ indicates coordination to framework oxygens O_fw_ in the proximity of two neighbouring charge-balancing Al in framework T-sites, *i.e.* 2Al) and redox-active Z[Cu(ii)OH] complexes (where Z denotes bonds to O_fw_ next to an isolated Al in a framework T-site, *i.e.* 1Al) preferentially located in the 8r^11^ and identified as the dominant structural component in high-loading Cu-CHA with Si/Al ∼ 13.^24,28^

Aiming for a comprehensive experimental exploration of the composition effects on Cu-speciation in activated Cu-CHA, we synthesized a systematic series of Cu-CHA samples, with Si/Al and Cu/Al ratios in the ∼5–29 and ∼0.1–0.6 ranges, respectively. A detailed description of the synthesis procedures and of the compositional characterization of all catalysts is reported in the ESI Section 1.1.† We explore by *in situ* XAS the composition-dependent Cu-speciation of such catalysts during thermal treatment in He flow from RT to 400 °C (He-activation), in order to differentiate between the different reducibilities of Cu-sites and gain deeper insights into the so-called self-reduction process in Cu–zeolites.^29–32^

Indeed, even after decades of research, the formation of large Cu(i) populations during the thermal treatment of Cu(ii)-exchanged zeolites under vacuum or inert gas flow, thus in the absence of any manifest reducing agent, is still puzzling the scientific community.^29–32^ The self-reduction process is now gathering renewed interest in the context of the direct methane to methanol conversion on Cu–zeolites, where a mechanism is envisaged that requires the reaction of Cu(i) sites with O_2_ to form CH_4_-activating Cu-oxo species.^32^

Multivariate curve reconstruction (MCR) analysis of temperature-dependent XANES in combination with multi-component EXAFS fits allowed us to single out the XAS signatures of individual Cu-species formed in the CHA cages and achieve unprecedented quantitative information on composition *versus* Cu-speciation relationships. The composition-dependent reducibility and the complex nature of the ZCu(i) species resulting from self-reduction of Z[Cu(ii)OH] complexes are further investigated using FTIR spectroscopy of adsorbed N_2_. XAS and FTIR results reported hereafter merge into a consistent picture, adding novel traits of complexity to the state-of-the-art understanding of composition- and conditions-driven Cu-speciation in Cu-CHA.

## 2 Results and discussion

### 2.1 *In situ* XAS during He-activation on Cu-CHA catalysts at different compositions

As demonstrated by recent literature on Cu–zeolite catalysts,^1–3,7,21,24,33^ *in situ* Cu K-edge XAS is an ideal technique to access direct information on the average electronic properties and coordination environment of the metal centres hosted in the zeolite cages.^34–37^ In order to track the dynamic rearrangements of Cu ions as a function of the environmental parameters, we collected *in situ* XANES during He-activation from 25 °C to 400 °C (flow rate 100 ml min^–1^; heating rate 5 °C min^–1^; XANES scan duration ∼6 min) for six Cu-CHA samples with Si/Al ratios of ∼5, 15, 19, 29 and Cu/Al ratios from ∼0.1 to ∼0.6. Experimental details on the gas-flow setup and XAS acquisition parameters are reported in the ESI Section 1.2.†

**Fig. 1:**
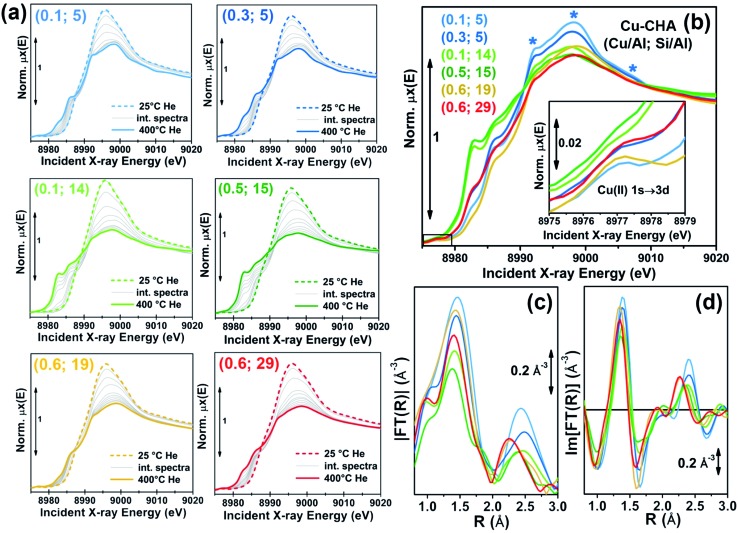
(a) *In situ* XANES of Cu-CHA catalysts with different compositions (different samples are denoted with (Cu/Al; Si/Al) labels) during dehydration under He flow from 25 °C to 400 °C, heating rate 5 °C min^–1^. (b) Comparison between XANES spectra of He-activated Cu-CHA samples with different compositions, collected at 400 °C. The inset shows a magnification of the weak pre-edge peak mostly deriving from the dipole-forbidden 1s → 3d transition in Cu(ii) sites. (c, d) Magnitude (c) and imaginary parts (d) of the *k* ^2^-weighted, phase uncorrected, Fourier transformed EXAFS spectra (*k*-range 2.4–12.4 Å^–1^) of He-activated Cu-CHA samples with different compositions, collected at 400 °C. Colour code for all of the panels: dashed thick lines: hydrated sample at 25 °C; solid thick lines: dehydrated sample at 400 °C; grey thin lines: intermediate spectra.

Fig. 1a shows the evolution of *in situ* XANES of the investigated Cu-CHA catalysts during He-activation from 25 °C to 400 °C. The XANES features follow similar trends throughout the whole series of samples, in agreement with previous studies by us^19,24^ and others.^21^ The spectroscopic features emerging during activation can be qualitatively interpreted on the basis of the abundant literature on Cu K-edge XANES in metal-exchanged zeolites^1–3,7,19,22,24,25,29,30,38–45^ and Cu active sites in biologically-relevant systems.^46–48^ In particular, a progressive decrease of the intense white-line peak at ∼8996 eV, characteristic of mobile five/six-coordinated Cu aquo-complexes,^24,49–52^ is observed as the activation proceeds, paralleled by the development of rising-edge peaks in the 8985–8987 eV range, reflecting an increase in the population of four/three-coordinated Cu(ii) sites.^29,31,47,53^ From ∼230 °C upwards, the self-reduction occurs in all of the samples, which can be tracked by the development of XANES features in the 8981–8985 eV range typical of Cu(i) sites in non-linear, low-coordination-number environments.^29,38,42,46,54,55^

We also collected higher-quality *in situ* XAS spectra of the whole sample series upon stabilization at 400 °C in He. The acquisition time was adjusted to achieve a satisfactory S/N ratio in the EXAFS region (the corresponding *k*-space *k* ^2^ *χ*(*k*) EXAFS spectra are reported in the ESI, Section 5.1†), enabling quantitative fitting of the resulting EXAFS spectra, see the ESI Section 1.2.2 for details.† Fig. 1b–d compare the final states reached in He flow at 400 °C for all samples, in both the XANES (Fig. 1b) and EXAFS region (Fig. 1c and d). A progressive modulation of the XAS features as a function of the composition can be appreciated. The rising-edge and post-edge peaks in the XANES occur at very similar positions in the whole sample series, although their relative intensity is different depending on the composition. The reducibility (relative abundance of Cu(i) species at 400 °C) of the samples seems to be mostly determined, in a non-monotonous way, by the Si/Al ratio. A lower reducibility is observed for both the Si/Al = 5 and Si/Al = 19 and 29 samples, although the dominant dehydrated Cu(ii) species appear to be different in the two cases. In particular, the XANES for He-activated Cu-CHA at Si/Al = 5 shows a more intense and structured white line region, with well-resolved peaks at ∼8998 and ∼8992 eV, and a broad post-edge peak in the 9005–9010 eV range (blue asterisks in Fig. 1b), in agreement with the XAS spectra reported by Paolucci *et al.* ^21^ for a Cu-CHA catalyst with a similar composition (Cu/Al = 0.08; Si/Al = 5). Conversely, the samples with Si/Al = 19 and 29 show broader and less pronounced white-line peaks, as well as a smoother post-edge region. The catalysts with intermediate Al content, Si/Al = 14 and 15, are characterized by very pronounced Cu(i) XANES features, pointing out the presence of a majority of Cu(i) sites at 400 °C in He, irrespective of Cu-loading.

The reducibility trends qualitatively identified throughout the series of samples are also supported by the modulations in the intensity of the weak pre-edge peak at *ca.* 8977 eV, mostly deriving from the 1s → 3d transition, which unambiguously fingerprints the presence of d^9^ Cu(ii) ions in the catalysts^38,46,47,56^ (see Fig. 1b, inset). Such a peak is clearly visible for all the investigated samples except for the Si/Al ∼ 14 and 15 ones, where it appears almost completely smeared at both high (Cu/Al = 0.5) and low (Cu/Al = 0.1) Cu-loading.

For all of the He-activated samples, two well defined maxima are distinguishable in the phase-uncorrected FT-EXAFS spectra (Fig. 1c and d), occurring in the 1.4–1.5 Å and 2.2–2.4 Å ranges. Based on the DFT-assisted EXAFS analysis previously performed on a Cu-CHA sample with Cu/Al ∼ 0.44 and Si/Al ∼ 13,^24^ we can safely assign the first maximum to single scattering (SS) paths involving extra-framework (O_ef_) and framework (O_fw_) O atoms in framework-coordinated Cu-species. Remarkably, the composition-induced modulation in the first-shell intensity correlates with the reducibility as evaluated from XANES. Indeed, samples with Si/Al = 5 and Si/Al = 19 and 29 show higher first-shell intensities, suggesting a major population of Cu(ii) sites characterized by three/four-fold coordination to O ligands. Conversely, a lower first-shell intensity is observed at Si/Al 14 and 15, corresponding to a higher relative fraction of two-fold coordinated Cu(i) sites.

Based on several recent XAS studies on Cu-CHA,^3,21,22,24,43^ we assign the principal contribution in the second maximum of the FT-EXAFS spectra to SS paths involving second-shell Al/Si T-atoms of the zeolite framework (T_fw_). Notably, this characteristic EXAFS feature has been previously employed to discriminate between framework-interacting Cu-sites and ammonia-solvated mobile Cu-complexes in *operando* NH_3_-SCR studies on Cu-CHA.^3,21,22^ We observe a clear composition-driven trend in both intensity and position (more evident from looking at the imaginary part of the FT-EXAFS spectra in Fig. 1d) of the second-shell EXAFS peak. As the Si/Al ratio increases from 5 to 29, the peak progressively shifts towards lower *R*-values, evidencing an average shortening in Cu–T_fw_ bond distances (see also Section 2.4 for quantitative analysis). The intensity of the second-shell peak is mostly determined by the Si/Al ratio; a minimum is found in correspondence of the Si/Al = 14 and 15 samples, according to the following trend: Si/Al = 5 > 19 and 29 > 14 and 15.

Interestingly, the highest reducibility at Si/Al ∼ 15 is accompanied by damped and broadened second shell peaks, possibly connected with a higher heterogeneity in Cu(i) siting (see below, Sections 2.4 and 2.5). The higher second-shell intensity observed for Si/Al = 5 is in good agreement with the presence of a major contribution from 2Al Z_2_Cu(ii) sites. For these Cu-sites, synchrotron radiation powder XRD^10,11^ and DFT-optimized geometries previously reported in the literature,^21,24,28^ evidence two almost degenerated Cu–Al and Cu–Si SS paths at ∼2.8 Å contributing to the second maximum in the FT-EXAFS (please note that Si and Al atomic neighbours are not distinguishable by EXAFS, nor by XRD, due to the very similar scattering amplitude). Conversely, for the case of 1Al Z[Cu(ii)OH] sites, only one Al neighbour at ∼2.7 Å is predicted, which would result in a lower second-shell peak in the FT-EXAFS spectra.

The qualitative observations presented so far will be rationalized in the following sections by combining multivariate analysis of the temperature-dependent XANES dataset shown in Fig. 1a and multi-component fitting of the EXAFS spectra reported in Fig. 1c and d, together with simulated Cu K-edge XANES spectra for DFT-optimized model structures of the different identified Cu-species/sites.

### 2.2 Multivariate analysis of temperature-dependent *in situ* XANES

As highlighted in the previous section, qualitative analysis of the *in situ* XAS dataset during He-activation as shown in Fig. 1 strongly supports the presence of a common set of Cu-species, occurring with different relative abundance as a function of the temperature *T* and composition (Cu/Al; Si/Al). Thus, normalized experimental XANES spectra at each temperature and composition, *μ* ^exp^(Cu/Al; Si/Al, *T*, *E*), can be described as linear combinations (LC) of a limited number *N*_pure_ of reference XANES spectra of pure Cu-species/sites, *μ*pure*i*(*E*), with composition- and temperature-dependent weights *w*pure*i*(Cu/Al; Si/Al, *T*), according to eqn (1):
1

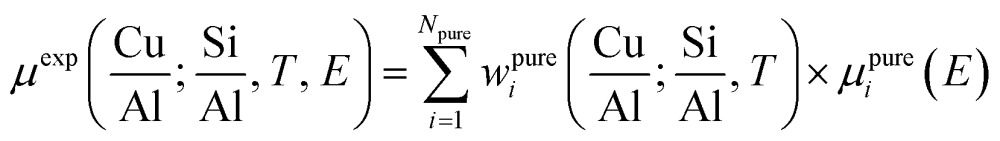




XANES linear combination fit (LCF) analysis has been established as a powerful tool for disentangling the different contributions in complex multi-component spectral series, and successfully applied to rationalize Cu-speciation during *in situ*/*operando* experiments on Cu-based catalysts.^22,33,40,57^ Nonetheless, a bottleneck in the effective application of this method is the availability of a proper set of reference spectra.

It is now well known that under certain conditions, *e.g.* for the as-prepared pre-catalyst at RT in air or during low-temperature SCR,^19–23^ the Cu cations in Cu-CHA are efficiently solvated by H_2_O or NH_3_, yielding mobile aquo or amino complexes that scarcely interact with the zeolite framework. In such conditions, molecular complexes in solution can be readily used as references in LCF analysis, yielding excellent results in the determination of Cu-speciation.^3,22^ However, when framework-interacting Cu-species are formed, the selection of a suitable set of references is not straightforward, due to the unusual coordination configurations. Intriguingly, these resemble the low-coordination/low symmetry environments of the active sites in metallo-proteins,^48,58,59^ and are difficult to replicate synthetically. With this respect, we have attempted to reproduce the XANES spectra of the He-activated Cu-CHA samples reported in Fig. 1b using conventional LCF analysis with a series of relevant experimental references, namely hydrated Cu(ii), [Cu(ii)(NH_3_)_4_]^2+^, CuO and Cu_2_O. However, LCF resulted in an unsatisfactory reproduction level of the experimental data, with *R*-factor values in the 0.05–0.1 range, depending on catalyst composition. Computational analysis has played a key role in identifying major Cu-species in dehydrated Cu-CHA, but despite the enormous progress in DFT-assisted XANES simulations,^33,34,60–63^ in most of the cases the line-to-line agreement between experimental and theoretical spectra, required to directly employ theoretical references for XANES LCF analysis, is still difficult to achieve.

To address this challenge, we explored a novel route in the analysis of the multi-composition temperature-dependent dataset reported in Fig. 1, exploiting multivariate curve resolution (MCR) techniques^64–66^ to deduce XANES spectra of pure Cu-species directly from the available experimental data. These chemometric methods are helpful in resolving mixtures, once the number of pure constituents, *N*_pure_, is independently determined, by estimating their response profiles and their concentrations (in our case normalized XANES spectra *μ*pure*i*(*E*) and composition- and temperature-dependent weights *w*pure*i*(Cu/Al; Si/Al, *T*), see eqn (1) and ESI Section 1.4†). The proliferation of *operando* XAS spectroscopy, involving the time-efficient collection of big datasets during dynamic process-relevant conditions, is triggering an increasing demand for automated data analysis strategies. In this context, MCR methods, originally developed and widely employed by the analytical chemistry community, have recently emerged as versatile tools in the rationalization of time-resolved *in situ*/*operando* spectroscopic data in heterogeneous catalysis, and a few examples of application to XAS studies can be found in the literature.^67–71^

Notably, once a series of *μ*pure*i*(*E*) spectra is extracted by MCR-ALS, DFT-assisted XANES simulations represent a powerful tool to qualitatively guide *a posteriori* the assignment of each component to specific metal environments (see below).

The first step in our analysis involved the determination of *N*_pure_ in the multi-composition temperature-dependent XANES dataset in Fig. 1a. To this aim, we performed principal component analysis (PCA) of the *in situ* XANES series (He-activation from 25 to 400 °C) for each catalyst composition.

PCA has been performed assuming that any systematic thermal dependence of the XANES signal is negligible with respect to the Cu-speciation-related contributions to the experimental variance (see the ESI Section 2.1† for details and justification of this hypothesis). From inspection of the Scree plots and quantitative analysis using the Malinowski *F*-test (5% significance level) and IND-factor,^64^ we identified an average number of pure species 〈*N*_pure_〉 = 5 ± 1 (see also the ESI Section 2†). Subsequently, we employed an MCR approach, based on the alternating least square (ALS) method,^65,66^ to extract chemically meaningful spectra and concentration profiles of pure Cu-species/sites during the He-activation process. MCR-ALS analysis was performed using the GUI (Graphical User Interface) developed by Tauler and co-workers,^72^ see the ESI Section 1.4 for details.†

Being aware that the selection of *N*_pure_ could significantly influence the MCR-ALS results, we systematically repeated the analysis for *N*_pure_ values in the 3–6 range. The reconstruction results obtained by upsizing/downsizing the component space around *N*_pure_ = 5 are reported in the ESI Section 3.† These were carefully assessed in terms of (i) convergence of the MCR-ALS algorithm, (ii) ability to reproduce all the characteristic XANES features (local maxima in the theoretical *μ*pure*i*(*E*) curves) identified in the experimental dataset, (iii) trends in the *R*-factor values for reconstruction, (iv) presence of overlapping contributions in the derived concentration profiles, and (v) chemical and spectroscopic meaningfulness of the derived scenarios. All these indicators strongly support the fact that the number of pure components identified by statistical analysis, *N*_pure_ = 5, represents an optimal value within the available data quality and time-resolution, as also confirmed by the low *R*-factor values and residuals obtained for the adopted five-component model (see below, Section 2.3 and ESI Section 1.4.2 and 3.2†).

**Fig. 2:**
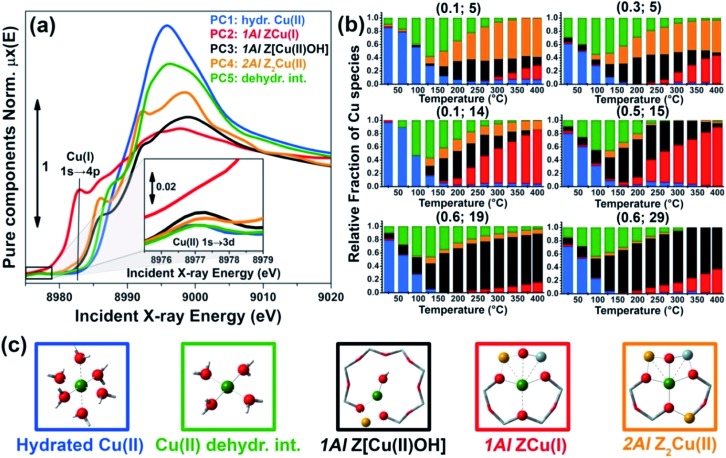
Results of MCR-ALS analysis of global temperature-dependent XANES dataset collected for six Cu-CHA samples with different compositions during He-activation from 25 to 400 °C (Fig. 1a), assuming *N*_pure_ = 5. (a) XANES spectra of pure components *μ*pure*i*(*E*) derived from MCR-ALS. The inset reports a magnification of the Cu(ii) 1s → 3d transition region in the theoretical spectra. (b) Temperature-dependent abundance of pure species, *w*pure*i*(Cu/Al; Si/Al, *T*), in each of the catalysts (bars have the same colours as the corresponding spectra in panel (a)). (c) Proposed assignment of the five pure components to specific Cu-species/sites formed in the Cu-CHA catalyst as a function of composition and activation temperature, using the same colour code as in parts (a) and (b). Blue (PC1): mobile Cu(ii)-aquo-complexes [Cu(ii)(H_2_O)_*n*_]^2+^/[Cu(ii)(H_2_O)_*n*–1_(OH)]^+^ with *n* = 6; green (PC5): Cu(ii) dehydration intermediate, possibly represented by mobile [Cu(ii)(H_2_O)_*n*_]^2+^/[Cu(ii)(H_2_O)_*n*–1_(OH)]^+^ complexes with *n* = 4; black (PC3): 1Al Z[Cu(ii)OH] sites in their oxidized form; red (PC2): 1Al ZCu(i) sites in their reduced form, resulting from self-reduction of 1Al Z[Cu(ii)OH] species; orange (PC4): 2Al Z_2_Cu(ii) sites. Atom colour code: Cu: green; H: white; O: red; Si: grey; Al: yellow.

Fig. 2 summarizes the results from the MCR-ALS analysis of He-activation in Cu-CHA as a function of temperature and catalyst composition. The five theoretical XANES spectra of pure components *μ*pure*i*(*E*) are shown in Fig. 2a while the bar plots in Fig. 2b report their corresponding temperature-dependent concentration profiles for each compositional point. By matching the characteristic XANES features of each *μ*pure*i*(*E*) spectrum and the evolution trends for their weights *w*pure*i*(Cu/Al, Si/Al; *T*) with the rich experimental background currently available on Cu-CHA, we were able to safely assign each pure component to the Cu-species depicted in Fig. 2c, as we will discuss hereafter. Further support to the assignment will be derived from comparisons with the experimental Cu K-edge XANES spectra of selected reference compounds and by DFT-assisted XANES simulations based on the models reported in Fig. 2c.

A first clue to identify the Cu oxidation state in each of the retrieved pure components is provided by the 1s → 3d pre-edge peak in the theoretical pure spectra *μ*pure*i*(*E*) (see Fig. 2a, inset). The peak is clearly present in all of the *μ*pure*i*(*E*) curves except in the *μ*pure2(*E*) XANES for the PC2 component (red line in Fig. 2a). Hence, this latter component can be unambiguously assigned to a d^10^ Cu(i) species, whereas the other four components correspond to Cu(ii) moieties.

Considering the characteristic shape of the XANES spectrum, the PC1 component is easily assigned to mobile Cu(ii)-aquo-complexes, namely [Cu(ii)(H_2_O)_*n*_]^2+^ or [Cu(ii)(H_2_O)_*n*–1_(OH^–^)]^+^ with *n* = 6. For all of the probed compositions, these fully hydrated Cu(ii) ions represent the dominant component at the beginning of the activation process, and their concentration drops rapidly to values <10% total Cu as the temperature reaches ∼150 °C. Further support to such assignment and, in general, to the spectroscopic reliability of the theoretical XANES derived by MCR-ALS, is provided by the comparison between the *μ*pure1(*E*) curve and the reference XANES for an aqueous solution of Cu(ii) acetate, see the ESI Section 4.1.†

The decrease in the relative fraction of fully hydrated Cu(ii) is paralleled by the transient growth in the contribution of a different component (PC5), whose concentration peaks in the 100–130 °C range for all of the samples. The correspondent *μ*pure5(*E*) spectrum is characterized by a lower intensity and a broader shape in the XANES white-line region. A shoulder becomes evident in the edge-rising region at ∼8987 eV. We tentatively assigned such a component to a four coordinated, pseudo square-planar Cu(ii) ‘dehydration intermediate’, based on the similarity of the corresponding MCR-ALS XANES to the spectra of the reference four-coordinated Cu(ii) species, *i.e.* Cu(ii)O and [Cu(ii)(NH_3_)_4_]^2+^ (see the ESI Section 4.1†). Mobile Cu(ii) complexes such as [Cu(ii)(H_2_O)_4_]^2+^/[Cu(ii)(H_2_O)_3_(OH)]^+^ or alternatively four-coordinated Cu(ii) moieties with mixed O_fw_/O_ef_ ligation could be envisaged. Remarkably, the contribution from this species is appreciable until 250–280 °C (albeit with longer tails in the Si/Al = 19 and 29 samples), which matches well the temperature threshold at which no more adsorbed molecular water was observed by FTIR on a Cu-CHA catalyst with Si/Al ∼ 13 and Cu/Al ∼ 0.44.^24^

The decay in the population of the dehydration intermediate described above paves the way to the actual birth of the fully dehydrated framework-interacting Cu-species. It is here that the impact of the catalyst composition on temperature-dependent Cu-speciation comes strikingly into play. Two Cu(ii) species start to form almost simultaneously after ∼130 °C, namely the PC3 and PC4 components, that we assign to the 1Al Z[Cu(ii)OH] and 2Al Z_2_Cu(ii) sites, respectively. Such an assignment is supported by a wealth of evidence. Firstly, the PC4 component dominates high-temperature Cu-speciation at Si/Al = 5, reaching at 400 °C relative fractions from ∼40 to ∼60% of total Cu depending on the loading. Conversely, it always represents a minor component (<20% total Cu) at both intermediate (Si/Al = 14, 15) and low (Si/Al = 19 and 29) Al content. Moreover, after 250 °C, the contribution from PC4 is substantially stable,‡
‡This is clearly observed for Si/Al = 5 catalysts, where 2Al Z_2_Cu(ii) sites are present in large abundance (>40% total Cu). In some of the other samples, where 2Al Z_2_Cu(ii) sites represent a minor component (<20% total Cu), their concentration for temperatures higher than 250 °C is subjected to fluctuations in the ±5% total Cu range, for which reliability is difficult to assess. Interestingly, such fluctuations in the 2Al Z_2_Cu(ii) population occur concomitantly to the rise in reduced 1Al ZCu(i) species and seem to correlate with a small transient increase in the concentration of hydrated Cu-complexes. whereas the PC3 population is progressively eroded in favour of PC2, the only Cu(i) component extracted by MCR-ALS. Spectroscopically, the correspondent *μ*pure3,4(*E*) spectra properly reflect the differences observed in the experimental data as a function of the composition. In particular, the *μ*pure4(*E*) curve associated to the 2Al Z_2_Cu(ii) sites optimally reproduces both the higher, highly structured, white line peak and the post-edge peak observed in the experimental XANES of He-activated samples at Si/Al = 5.

Finally, the PC2 contribution is straightforwardly attributed to the 1Al ZCu(i) sites, resulting from self-reduction of the 1Al Z[Cu(ii)OH] species. The correspondent *μ*pure2(*E*) spectrum shows all of the characteristic fingerprints of two-coordinated non-linear Cu(i) sites,^24,29,38,42,46,54,55^ with a prominent rising-edge peak developing from ∼8982 eV and a lower white line intensity with respect to the other Cu-species identified so far.

**Fig. 3:**
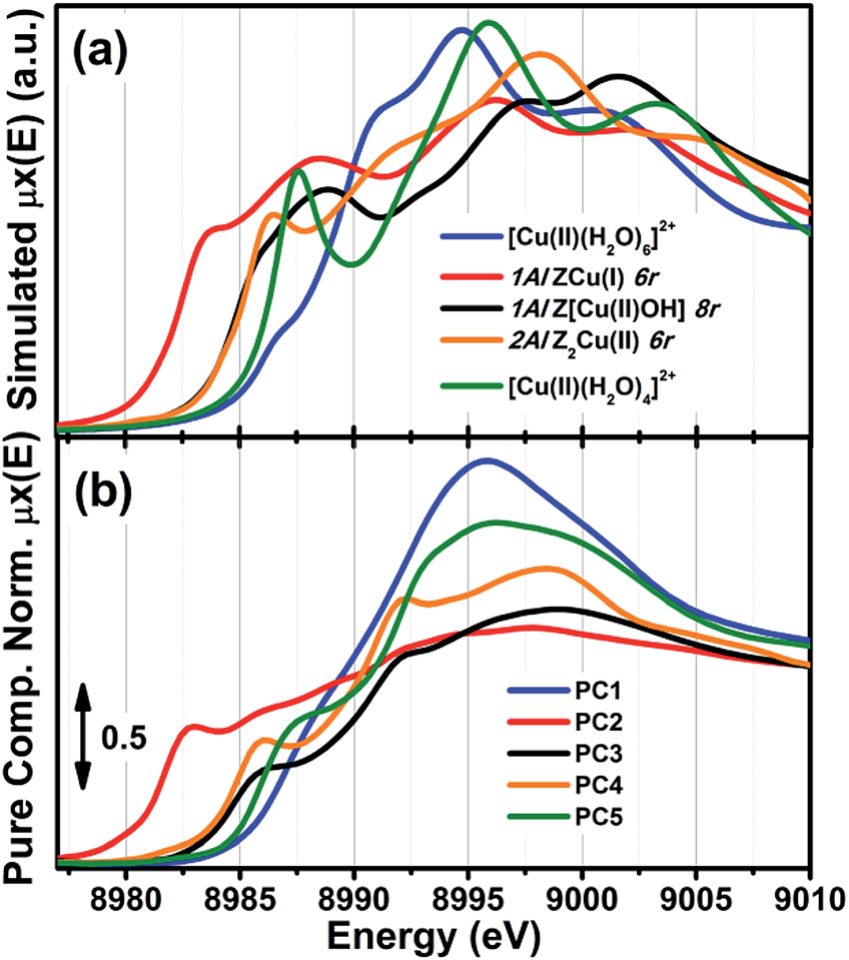
(a) Simulated Cu K-edge XANES spectra for the five DFT-optimized model structures reported in Fig. 2c and proposed for the interpretation of the MCR-ALS *μ*pure*i*(*E*) spectra. (b) *μ*pure*i*(*E*) spectra obtained from MCR-ALS analysis for comparison; same spectra as in Fig. 2a.

As anticipated, to further support these assignments we have simulated the Cu K-edge XANES spectra for all of the five model structures reported in Fig. 2c and proposed the interpretation of the MCR-ALS *μ*pure*i*(*E*) spectra; computational details on the strategy adopted to calculate the theoretical XANES for each DFT-model can be found in the ESI Section 1.5.† The five simulated XANES spectra and the correspondent *μ*pure*i*(*E*) curves from MCR-ALS analysis are compared in Fig. 3(a) and (b), respectively.

Focusing on the three framework-interacting Cu-species which dominate Cu-speciation in the He-activated catalysts, we observe how the simulated XANES curves properly reproduce the distinctive relative energy shifts (and partially, intensity ratios) of the rising-edge peaks for each Cu-site. The ZCu(i) model, in agreement with our assignment to PC2, results in prominent peaks significantly red-shifted and more intense with respect to the Z[Cu(ii)OH] and Z_2_Cu(ii) geometries. In line with the assignment to PC4, the Z_2_Cu(ii) site gives rise to a sharp peak at ∼8986 eV, two well defined maxima in the white-line region at ∼8992 eV and ∼8998 eV, and a broad post-edge peak around 9007 eV. The alternative Cu(ii) environment, Z[Cu(ii)OH], is associated to a broader rising-edge peak and less defined features in the white line region, globally shifted to higher energy, in qualitative agreement with the PC3 curve.

Theoretical spectra for fully- and under-coordinated Cu(ii) aquo-complexes are clearly distinguished from the ones of framework-interacting Cu-species due to the presence of high-intensity transitions in the 8990–8998 eV range, as observed in the MCR-ALS spectra for PC1 and PC5. Interestingly, the simulations evidence how, while decreasing the number of H_2_O ligands in the first Cu(ii) coordination sphere from six to four, a well-defined rising-edge peak develops at ∼8987 eV. The same behaviour is recognized while comparing the *μ*pure1(*E*) and *μ*pure5(*E*) curves, together with a slight blue-shift of the white-line peak for PC5, which is also reproduced in the simulated spectra for [Cu(ii)(H_2_O)_4_]^2+^ and [Cu(ii)(H_2_O)_6_]^2+^. Beside the simulations reported in Fig. 3a, we have also considered an intermediate [Cu(ii)(H_2_O)_5_]^2+^ model, aiming to improve the reproduction of the PC5 species. As can be observed in the ESI Section 4.2,† [Cu(ii)(H_2_O)_4_]^2+^ and [Cu(ii)(H_2_O)_5_]^2+^ substantially give rise to an equivalent set of peaks, but their relative intensity and energy position (especially in the white-line region) are affected by the number of H_2_O ligands. Although the similarity with four-coordinated reference compounds (see Fig. S10b†) suggests predominant ligation to 4 H_2_O ligands, simulations indicate that the *μ*pure5(*E*) spectrum could actually evidence a dynamic mix of four-, five-, and, possibly, even lower-coordinated Cu(ii) aquo complexes, which are however not readily resolvable within time- and energy-resolution of the available dataset. To resolve the additional complexity associated with the transition between mobile and framework-interacting Cu-species, we envisage additional studies employing molecular dynamics-assisted XANES simulations^73,74^ in combination with High Energy Resolution Fluorescence Detected (HERFD) XANES measurements, ensuring both higher energy resolution and improved time-resolution to accurately sample the 130–200 °C temperature range where the highest concentration of PC5 is observed.

### 2.3 Interpretation of the MCR-ALS results: novel elements of complexity affecting reducibility in Cu-CHA

From the MCR-ALS analysis discussed above, several insights into the impact of the catalyst composition on Cu-speciation during He-activation can be derived. At RT, Cu-speciation is largely dominated by mobile, fully-coordinated Cu aquo-complexes (always >60% total Cu). Nonetheless, small populations of partially dehydrated Cu(ii) species together with traces (<5% total Cu) of framework-interacting Cu(ii) and Cu(i) sites are already found at the beginning of the activation process. The birth of fully dehydrated Cu-sites occurs in all samples through the transient development of a common Cu(ii) ‘dehydration intermediate’, as described before. As shown in Fig. 2b, Cu-speciation for all of the He-activated catalysts at 400 °C can be described as a combination of redox-active 1Al sites (in their oxidized Z[Cu(ii)OH] or reduced ZCu(i) form) and redox-inert 2Al Z_2_Cu(ii) sites, in agreement with the picture proposed in the recent report by Paolucci *et al.*,^21^ and with the two reduction peaks observed for Cu-CHA during H_2_-TPR at ∼230 and 380 °C.^18,26^ Of note, the different redox properties of the 1Al and 2Al sites greatly favour their discrimination during thermal treatment under an inert atmosphere, since Cu(i) and Cu(ii) XANES features are much more readily discernible with respect to those of Cu(ii) sites with slightly different coordination geometries.

**Fig. 4:**
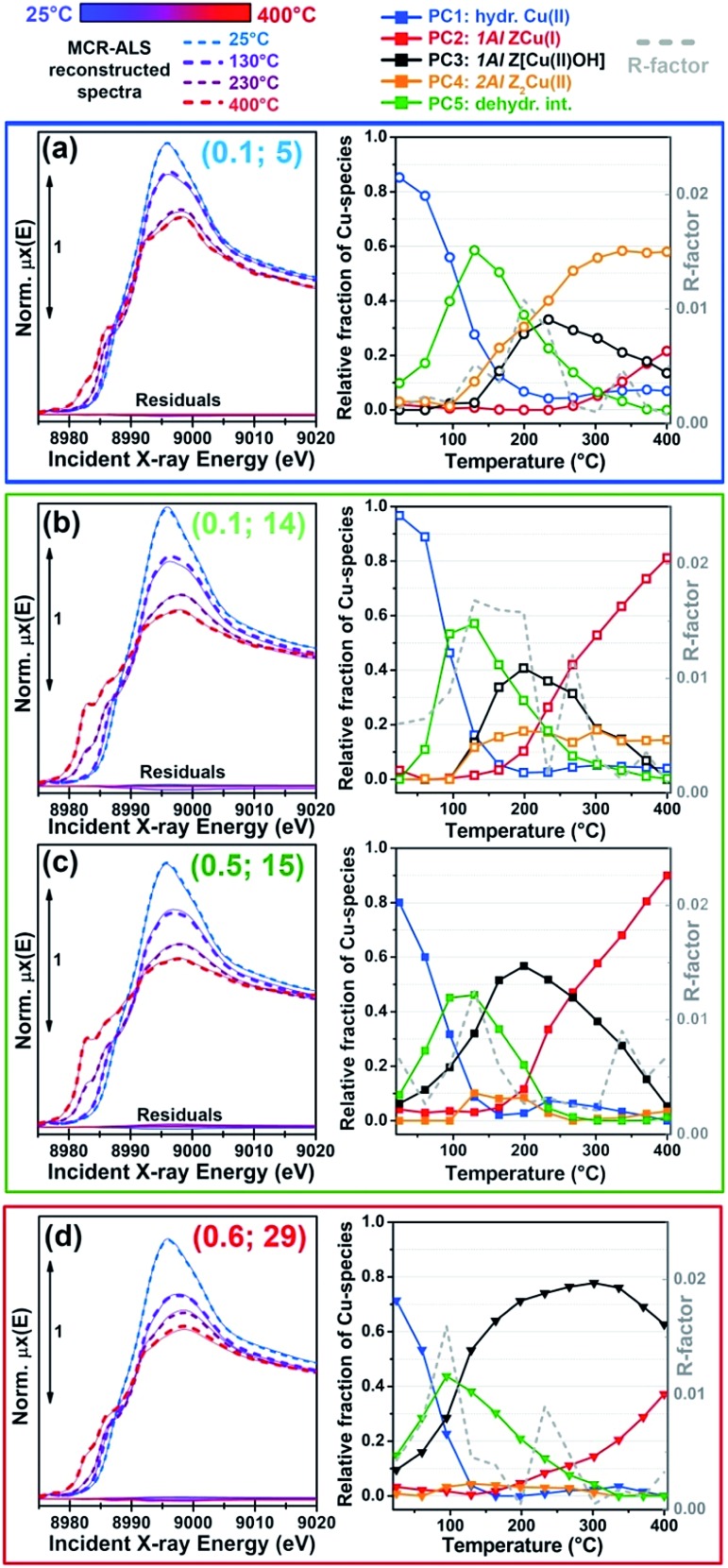
Detailed MCR-ALS results for the most representative compositional points: (a) Cu/Al = 0.1, Si/Al = 5; (b) Cu/Al = 0.1, Si/Al = 14; (c) Cu/Al = 0.5, Si/Al = 15; (d) Cu/Al = 0.6, Si/Al = 29. Left panels: comparison between experimental *in situ* XANES *μ* ^exp^ during He-activation (thin solid lines) and reconstructed spectra *μ* ^rec^ from MCR-ALS (thick dashed lines) at the four key temperatures of 25 °C, 125 °C, 230 °C, and 400 °C with the corresponding residuals (thick full lines). Right panels: concentration profiles for the five pure Cu-species/sites identified in our model as a function of the activation temperature (coloured symbols) and correspondent *R*-factor values (grey dashed lines).

Nonetheless, our analysis reveals novel elements of complexity, which will be addressed in more detail in the following sections. To this aim, Fig. 4 details the MCR-ALS results for the most representative compositional points, comparing experimental *μ* ^exp^(Cu/Al; Si/Al, *T*, *E*) and reconstructed *μ* ^rec^(Cu/Al; Si/Al, *T*, *E*) XANES at key temperatures during the He-activation (left panels) and reporting concentration profiles (right panels) for the five pure Cu-species identified by statistical analysis and assigned as described in Section 2.2. Reconstruction residuals for the selected temperatures and temperature-dependent *R*-factor values are also reported, demonstrating that the MCR-ALS procedure ensures an excellent reproduction of the experimental XANES dataset for all of the investigated samples (almost 100 spectra).

The highest deviations (peaks in *R*-factor *vs.* temperature profiles, which however are always well below 2%) are generally observed at the maximum population of the Cu(ii) dehydration intermediate or nearby, when different dehydrated Cu(ii) sites start to develop. As already discussed at the end of Section 2.2 and suggested by the MCR-ALS results for *N*_pure_ = 6 (see ESI Section 3†), the transition from mobile aquo complexes to framework-interacting species could occur *via* additional intermediate states (possibly composition-specific), which however are not resolvable within our experimental time-resolution and cannot be reliably singled out by MCR-ALS.

Paolucci *et al.* computed a Cu-site compositional phase diagram for activated Cu-CHA, predicting the relative fraction of 1Al and 2Al sites as a function of Si/Al and Cu/Al ratios.^21^ Such a diagram represents a milestone in Cu-CHA research. However, it involves some assumptions. In particular, it is derived assuming that the 2Al Z_2_Cu(ii) sites in the 6r represent the first framework sites to be populated by the cations during dehydration.

Under this hypothesis, only after all the available 2Al sites at a given Si/Al ratio are saturated, Cu is allowed to populate other kind of sites, primarily forming 1Al Z[Cu(ii)OH] complexes in the 8r. Similar assumptions were previously adopted by Bates *et al.* ^75^ to compute the Cu/Al ratio required to saturate the available 6r 2Al sites as a function of the Si/Al ratio in the parent zeolite. According to this model, catalysts with Cu-loadings below the saturation threshold would contain, after dehydration, only 2Al Z_2_Cu(ii) sites: for Si/Al = 5, 15 and 29, saturation is predicted at Cu/Al ≈ 0.24, 0.09, and 0.05 respectively.

However, the results reported in Fig. 4 reveal a more complex picture in relation with the population of the 2Al Z_2_Cu(ii) sites, particularly evident at intermediate Si/Al values. We note that for all of the investigated samples, 2Al Z_2_Cu(ii) and 1Al Z[Cu(ii)OH] sites (orange and black symbols in Fig. 4, respectively) start to form almost simultaneously during dehydration, which deviates from an ideal 2Al saturation scenario. Comparing the MCR-ALS results for the Si/Al = 5 catalysts with lower (Cu/Al = 0.1) and higher (Cu/Al = 0.3) loading, a saturation tendency is confirmed: due to the finite availability of 6rs hosting 2Al sites, at a higher Cu-loading a lower relative fraction of Z_2_Cu(ii) species is observed, counterbalanced by a larger population of reducible 1Al sites. Nevertheless, for the catalyst with Cu/Al = 0.1 and Si/Al = 14 (Fig. 4b), the final state at 400 °C is characterized by a substantial fraction of reduced ZCu(i) sites (∼80%), whereas the Z_2_Cu(ii) contribution reaches a maximum abundance of only ∼18%. Assuming saturation of the 2Al sites, this sample should be dominated by redox-inert 2Al sites due to the low Cu-loading (>90% total Cu according to the compositional phase diagram in ref. 21 and the saturation threshold evaluated by Bates *et al.* ^75^). Experimentally, this is however clearly not the case: MCR-ALS evidences only a relatively small increase in the abundance of 2Al sites with respect to the high-loading catalyst with a comparable Si/Al ratio (Cu/Al = 0.5; Si/Al = 15, Fig. 4c). This effect could be connected with high barriers for Cu migration towards 2Al sites, possibly trapping the cations into local energy minima at 1Al sites.

Another intriguing result emerging from MCR-ALS analysis is that the reducibility level for Cu-species formed at 1Al sites is clearly dependent on Al distribution, with the highest reducibility for Si/Al ∼ 15 (<10% Cu observed as 1Al Z[Cu(ii)OH] at 400 °C) and the lower reducibility at Si/Al = 19 and 29, with ∼70% and ∼60% of the 1Al sites still surviving in the oxidized form, respectively (see Fig. 2b and 4d). These results unambiguously demonstrate that self-reduction proceeds though a cooperative multi-step process involving proximal acid sites, the availability of which is ultimately determined by Al density and distribution in the zeolite. This is in line with previously suggested mechanisms in the literature.^21,30–32^ Plausibly, the process initiates with the thermally-driven reduction of Z[Cu(ii)OH] to ZCu(i), accompanied by the release of a hydroxyl radical, OH˙.^30–32^ The availability/spatial proximity of a reactive channel for the OH˙ radical might then determine the overall efficiency of the self-reduction process, and the final balance between Cu-species at 1Al sites in oxidized/reduced form observed in the He-activated catalysts. Consequently, Al-rich frameworks facilitate self-reduction. Nevertheless, in Cu-CHA (and, we might speculate, in general for d6r zeolites) the same condition also promotes large populations of redox-inert Z_2_Cu(ii) sites hosted in 6rs, lowering the average reducibility of the catalysts and blurring the overall picture. With respect to the Cu-loading impact on reducibility, when comparing concentration profiles for ZCu(i) on low- (Cu/Al ∼ 0.1) and high- (Cu/Al ∼ 0.5) loading Cu-CHA at almost equivalent Si/Al of ∼15, we observe very similar temperature onsets and rates for the self-reduction (see Fig. 4b and c). A slightly lower Cu(i) fraction is observed at 400 °C in the low-loading sample, but it excellently correlates with the small increase in the concentration of the Z_2_Cu(ii) sites. Hence, at optimal Si/Al values, Cu-loading seems to poorly affect the self-reduction efficiency, which can be conjecturally connected with the role of proximal Brønsted sites along parallel/alterative pathways efficiently affording ZCu(i) species. Within the time-resolution (∼6 min) of our XANES experiments, intermediate Cu(ii) species eventually formed along the transformation from Z[Cu(ii)OH] to ZCu(i) are not detected. From a structural point of view, it is however important to underline that the initial [Cu(ii)OH]^+^ complexes and other putative mono- and multi-meric intermediates proposed in the literature (*e.g.* [Cu(ii)O]^+^ (ref. 30 and 31) and mono-(μ-oxo) dicopper^7,32,76^) all retain a very similar tridentate coordination geometry for the Cu(ii) centres, expected to result in very similar XANES signatures. Hence, even assuming that such tridentate Cu(ii)-moieties do form and have a macroscopic lifetime in the catalyst before undergoing reduction to Cu(i) *via* extra-framework oxygen desorption,^77,78^ they could be easily assimilated in the same structural component as Z[Cu(ii)OH] in MCR-ALS analysis. [Cu(ii)OH]^+^ and [Cu(ii)O]^+^ complexes would also be virtually indistinguishable by EXAFS, due to the low scattering amplitude of H. Conversely, the formation of significant fractions of dicopper cores, possibly as a result of self-organization phenomena,^79^ should be traceable in the FT-EXAFS spectra *via* high-amplitude metal–metal contributions.

Finally, we note that for most of the samples, MCR-ALS analysis evidences a non-monotonous trend in the concentration profiles of hydrated Cu(ii)-species (PC1 in Fig. 4), showing a small peak in the high-temperature range (230–350 °C) which accounts for up to 5% total Cu increments in the relative fractions of fully hydrated Cu(ii). Due to the reduced entity of the variations and the absolute weakness of the contribution from this structural component in the high-temperature range, it is difficult to conclusively establish the physico-chemical meaningfulness of the effect. However, it is interesting to note that the temperature position of the peak correlates with the initial rise in the ZCu(i) component, supporting the fact that the initial steps in the pathway(s) to self-reduction involve the formation of small amounts of H_2_O. These traces of water could transiently re-solvate and mobilize a small fraction of Cu sites: for samples Cu/Al = 0.5; Si/Al = 15 and Cu/Al = 0.6; Si/Al = 29, the increase in the relative fractions of hydrated Cu(ii) apparently occurs at the expense of the 2Al Z_2_Cu(ii) sites (see Fig. 4c and d).

### 2.4 Structural analysis of 1Al and 2Al Cu-sites by multi-component EXAFS fits

Aiming for a further validation of the composition–speciation relationships discussed above and for a deeper structural characterization of the local coordination environment of the Cu ions hosted at 1Al and 2Al sites, we performed quantitative analysis of the *in situ* EXAFS spectra collected at 400 °C after dehydration in He (experimental data reported in Fig. 1c and d).

Here, singling out the contributions from the different Cu-sites is not straightforward. Unconstrained multi-component fits would result in an excess of strongly correlated free parameters, definitely yielding unreliable results. To obtain a robust set of constrains, we carried out a preliminary fitting step by selecting the compositional points characterized by the purest Cu-speciation at 400 °C (see also the ESI Section 5†), showing dominant contributions from each of the framework-interacting Cu-species evidenced by MCR-ALS, *i.e.* 1Al sites in their oxidized Z[Cu(ii)OH] or reduced ZCu(i) form and 2Al Z_2_Cu(ii) sites.

Importantly, at this stage, we also examined alternative environments for ZCu(i) and Z_2_Cu(ii), based on the DFT models obtained in our previous study,^24^ and already employed for the XANES simulations reported in Fig. 3. ZCu(i), initially formed from the reduction of Z[Cu(ii)OH] moieties in the 8r, could either remain in the 8r plane, or migrate to the energetically-favoured site in the 6r.^11,13^ The two locations are associated to different Cu local environments (especially in the second coordination sphere, see Table S5, ESI Section 5.3†), which might result in rather similar XANES signatures^24^ but different EXAFS features in the 2–3 Å range. Here, EXAFS analysis identifies the 6r site as the most likely configuration, but also evidences an increased structural disorder accompanying the self-reduction process. Indeed, both models resulted in a lower fit quality and higher Debye–Waller (DW) factors with respect to the case where the Cu(ii) oxidation state is largely dominant, either in the form of 1Al Z[Cu(ii)OH] or 2Al Z_2_Cu(ii) species. Notably, Göltl *et al.* ^80^ have recently modelled by *ab initio* molecular dynamics simulations the movements of bare Cu(i) and Cu(ii) cations in Cu-CHA at 300 K. The authors highlight how, already at RT, the local coordination of the Cu cations changes significantly, with larger movements for Cu(i), predicted to be more weakly bound to the framework with respect to Cu(ii). The possible compresence of a favoured docking site in the 6r with a secondary contribution from 1Al ZCu(i) sites in the 8r, together with the enhanced mobility of Cu(i) at 400 °C, is in excellent agreement with the blurring effect emerging for our EXAFS analysis. The co-existence of two different sites for Cu(i) in the 6r and 8r was also pointed out in a very recent quasi-simultaneous powder XRD/XANES study.^81^ Further insights on this point will be provided by *in situ* FTIR of adsorbed N_2_, as discussed in Section 2.5.

With respect to the 2Al Z_2_Cu(ii) 6r sites, within the limits imposed by Löwenstein’s rule,^82^ two possible configurations exist, depending on the siting for the two charge-balancing Al atoms within the 6r. In particular, –Al–Si–Al– and –Al–Si–Si–Al– linkages result in different Cu(ii) local environments: in the latter case, Cu is shifted towards the centre of the 6r, adopting a less distorted four-fold coordination to O_fw_. EXAFS analysis indicates that this –Al–Si–Si–Al– configuration optimally describes the 2Al Z_2_Cu(ii) site, yielding a significantly lower fit *R*-factor with respect to the –Al–Si–Al– geometry (see Table S6, ESI Section 5.3†). This result provides experimental support to recent DFT calculations predicting a more efficient stabilization of bare Cu(ii) sites in the 6r with –Al–Si–Si–Al– linkages.^12^

**Fig. 5:**
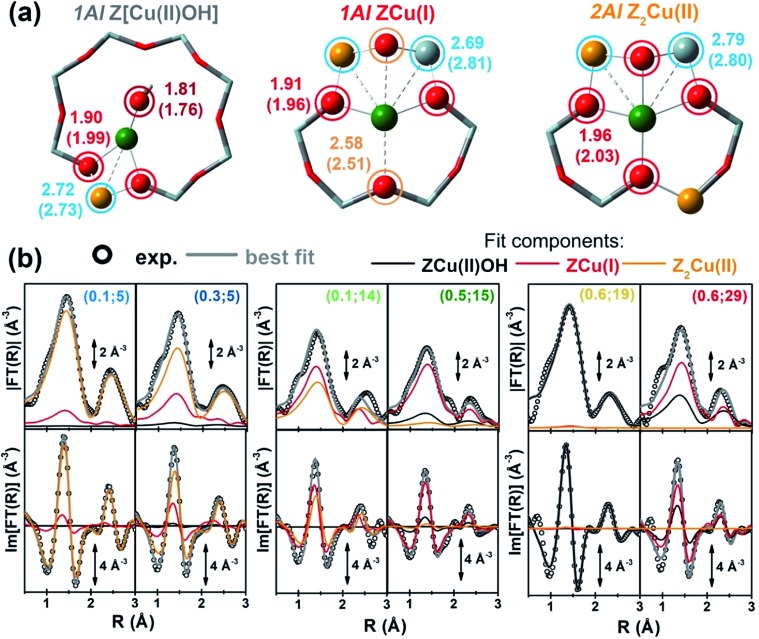
(a) DFT-optimized structural models for framework-interacting Cu-species dominating Cu-speciation in He-activated Cu-CHA at 400 °C, namely 1Al Z[Cu(ii)(OH)], 1Al ZCu(i), and 2Al Z_2_Cu(ii) sites. The atoms located at the first and second coordination shell of Cu are shown in ball-and-stick mode (atom colour code: Cu, green; H, white; O, red; Si, grey; Al, yellow) and highlighted with coloured circles indicating the different coordination shells included in the EXAFS fitting model (Cu–O_ef_: wine; Cu–O_fw_: red; Cu–O′_fw_: orange; Cu–T_fw_: blue). For each shell, the average bond distances from Cu refined by mono-component EXAFS fits are reported in Å, whereas the correspondent starting values from DFT are reported in parentheses. (b) Comparison between experimental (black circles) and best fit (light grey thick lines) FT-EXAFS spectra (top panels: magnitude, bottom panels: imaginary part) obtained from multi-component fits on the whole Cu-CHA sample series; for each sample, the weighted contributions from the three dehydrated Cu-species included in the fitting model are also reported as thin solid lines (1Al Z[Cu(ii)OH]: grey, 1Al ZCu(i): red; 2Al Z_2_Cu(ii): orange). The correspondent experimental and best fit *k* ^2^ *χ*(*k*) curves in *k*-space can be found in the ESI, Section 5.5.† In part (b), sample composition is indicated by (Cu/Al; Si/Al) labels.

Mono-component EXAFS fits provided us with experimentally-optimized geometries for the three framework-interacting Cu-species expected to dominate Cu-speciation in He-activated Cu-CHA (see Fig. 5a), setting the scene for a multi-component fitting protocol extended to the whole series of catalysts. To this aim, we calculated three sets of EXAFS paths including all the SS paths contributing in the 1.0–3.2 Å range for the Cu-sites shown in Fig. 5a. For each geometry, we fixed bond distances to the best-fit values obtained in the correspondent mono-component fit, and set a global *S*_0_^2^ to the ideal value of 1. Hence, for each catalyst composition, we fitted DW factors for the relevant shell of atomic neighbours, and, most importantly, relative fractions *A*_i_ for each component, i = Z[Cu(ii)OH], ZCu(i), or Z_2_Cu(ii). The results of the multi-component EXAFS fits are summarized in Fig. 5b and Table 1. For all catalysts, we were able to achieve a very good level of reproduction of the experimental EXAFS spectra by combining the three structural components singled out by MCR-ALS. The DW values refined for the different sub-shells of atomic neighbours are consistent with those found in previous studies, falling in the typical expectation ranges for high-temperature data collection on complex, multi-component systems.

**Table 1:** Results from multi-component EXAFS fits of the He-activated state at 400 °C for the whole multi-composition sample series. Fits are performed in the *k*-range (2.4–11.0) Å^–1^ and *R*-range (1.0–3.2) Å, resulting in a number of independent parameters πΔ*R*Δ*k*/2 > 12.^34^ For all of the fits, geometries of 1Al Z[Cu(ii)OH], 1Al ZCu(i), and 2Al Z_2_Cu(ii) Cu-species (*i.e.* radial shift parameters Δ*R*_*i*_) have been fixed based on the results of mono-component fits on the ‘purest’ He-activated states (1Al Z[Cu(ii)OH] model for sample (0.6; 19), 1Al ZCu(i) in the 6r for sample (0.5; 15), 2Al Z_2_Cu(ii) in the 6r with Al–Si–Si–Al linkage for sample (0.1; 5); see the ESI Section 5.3), and *S*_0_^2^ has been set to unity

Composition (Cu/Al; Si/Al)	(0.1; 5)	(0.3; 5)	(0.1; 14)	(0.5; 15)	(0.6; 19)	(0.6; 29)
Fit *R*-factor	0.007	0.007	0.044	0.017	0.006	0.014
*N*_par_ (*N*_ind_)	8 (12)	8 (12)	8 (12)	8 (12)	8 (12)	8 (12)
Δ*E* (eV)	–5.8 ± 0.6	–6.0 ± 0.5	–7 ± 1	–7.0 ± 0.8	–3.2 ± 0.4	–3.8 ± 0.4
*A*_Z[Cu(ii)OH]_	0.0 ± 0.2	0.0 ± 0.1	0.0 ± 0.3	0.1 ± 0.2	1.0 ± 0.2	0.4 ± 0.1
*A*_ZCu(i)_	0.2 ± 0.1	0.4 ± 0.1	0.7 ± 0.1	0.8 ± 0.2	0.0 ± 0.1	0.6 ± 0.2
*A*_Z_2_Cu(ii)_	0.8 ± 0.2	0.6 ± 0.1	0.3 ± 0.1	0.1 ± 0.1	0.0 ± 0.1	0.0 ± 0.1
*σ*_O(ef)_^2^ (Å^2^)	0.007 ± 0.002	0.008 ± 0.002	0.008 ± 0.002	0.007 ± 0.002	0.007 ± 0.001	0.007 ± 0.001
*σ*_O(fw)_^2^ (Å^2^)	0.007 ± 0.001	0.007 ± 0.001	0.008 ± 0.001	0.009 ± 0.001	0.004 ± 0.001	0.007 ± 0.001
*σ*_T(fw)_^2^ (Å^2^)	0.011 ± 0.002	0.014 ± 0.003	0.010 ± 0.002	0.012 ± 0.002	0.014 ± 0.003	0.009 ± 0.001

The highest levels of structural disorder are still seen for the Si/Al = 14 and 15 samples. This is not surprising, due to the fickle nature of the Cu(i) component which dominates in these highly-reducible catalysts. For all of the other samples, *R*-factor values well below 2% are obtained, in line with the lower mobility of the Cu(ii) ions.

Of note, for all of the investigated samples, the second-shell peak in the FT-EXAFS spectra is properly reproduced solely considering Cu–T_fw_ contributions, in excellent agreement with the different environments predicted for the 1Al and 2Al sites shown in Fig. 5a. In general, the multi-component fits reported in Fig. 5b discourage a significant contribution of Cu–Cu paths in the second-shell region, *i.e.* in the 2.0–2.8 Å range of the phase-uncorrected FT-EXAFS. We cannot however exclude that such Cu–Cu scattering contributions could emerge at higher distances, where unfortunately the technique is not accurate enough. Further experiments focusing on high-temperature activation in O_2_ and employing enhanced data collection statistics to improve the S/N ratio in the high *k*-range would be needed to achieve conclusive results on the presence of diluted multimeric Cu-oxo moieties.

**Fig. 6:**
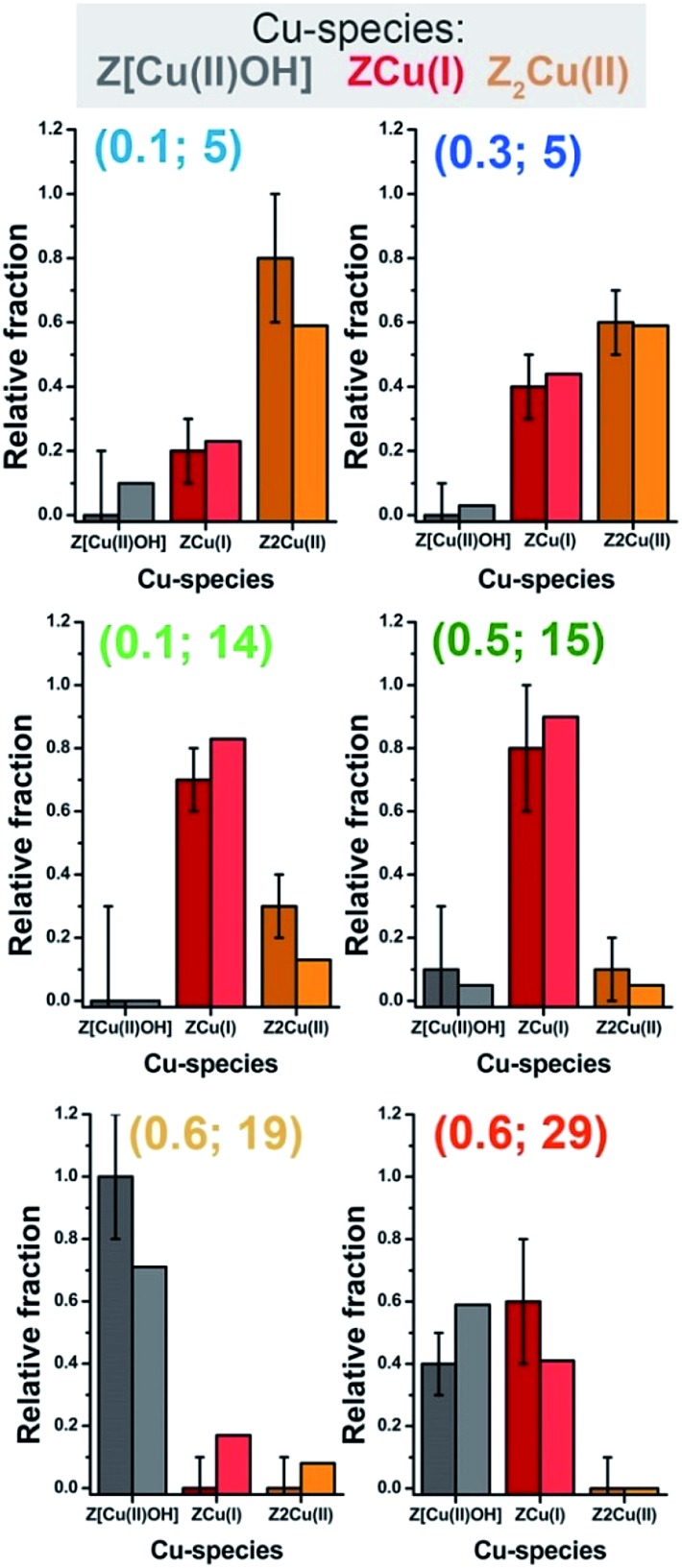
Relative fractions of framework-interacting Cu-species formed in He-activated Cu-CHA as a function of the catalyst composition (Z[Cu(ii)OH]: grey; ZCu(i): red; Z_2_Cu(ii): orange), evaluated from multi-component EXAFS fits (darker colours with error bars) and by MCR-ALS analysis of *in situ* XANES (lighter colours). Sample composition is indicated using (Cu/Al; Si/Al) labels.

In view of a cross-validation of the employed methods, Fig. 6 compares, as a function of the catalyst composition, the relative fractions of the Z[Cu(ii)OH], ZCu(i), and Z_2_Cu(ii) species in He-activated Cu-CHA at 400 °C evaluated from MCR-ALS XANES analysis and multi-component EXAFS fits. Within the EXAFS fitting errors, the two methods yield a substantially comparable Cu-speciation in the final He-activated state, corroborating the speciation–composition trends discussed above.

### 2.5 Reducibility trends and Cu(i) siting from *in situ* FTIR of adsorbed N_2_

*In situ* FTIR spectroscopy was employed as a complementary technique to corroborate the reducibility trend of Cu sites observed by XAS in the same set of Cu-CHA samples. In more detail, herein we discuss the spectra obtained using N_2_ as a probe molecule, on vacuum-activated samples. Spectra were normalized with respect to pellet thickness and Cu content, as detailed in the ESI Section 1.3.† N_2_ is a weakly interacting probe, enabling discrimination between similar adsorption sites due to the very low perturbation induced on the involved cations.^83–85^ Moreover, it selectively forms adducts (stable at liquid nitrogen temperature, ∼–160 °C) with Cu(i) sites,^38,86^ and it is widely accepted that vacuum activation is equivalent to treatment under inert flow (such as He), with respect to self-reduction.^19,31^ This means that the results described hereafter can be compared on a semi-quantitative ground to those discussed above for XAS.

**Fig. 7:**
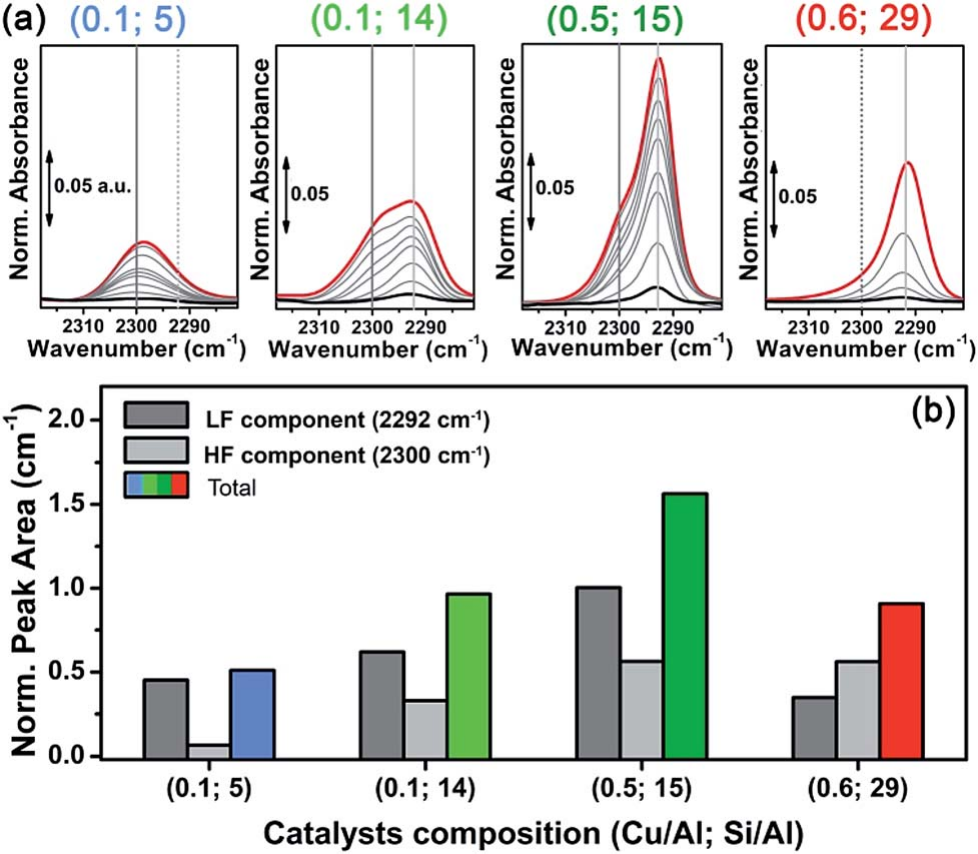
(a) Low temperature (∼–160 °C) normalized FTIR spectra of N_2_ dosed at increasing equilibrium pressure (from 10^–2^ to 5 Torr) on vacuum-activated Cu-CHA catalysts with different compositions, indicated by (Cu/Al; Si/Al) labels. Black, red and light grey curves refer to the lowest, highest and intermediate N_2_ coverage, respectively. (b) Normalized areas of the N_2_/Cu(i) IR peaks measured at the highest coverage, including normalized areas of LF and HF components calculated by spectral deconvolution.

N_2_ adsorption on a vacuum activated Cu-CHA sample with Cu/Al ∼ 0.44 and Si/Al ∼ 13 was reported by Giordanino *et al.*, and compared to the results obtained on Cu-beta and Cu-ZSM-5 with similar compositions.^86^ The spectra are in perfect agreement with those of the (Cu/Al = 0.5; Si/Al = 15) Cu-CHA sample studied in this work, clearly showing two components: a predominant band at ∼2292 cm^–1^ (light grey vertical line in Fig. 7a) with a shoulder centred at ∼2300 cm^–1^ (grey vertical line in Fig. 7a). This implies the presence of two significantly different Cu(i) sites, in terms of the cation local environment, in line with the report by Dědeček *et al.* who observed two distinct emission bands – assigned to Cu(i) ions in the 8r and 6r – on a set of natural and synthetic Cu-CHA zeolites.^87^

Interestingly, the relative intensity of the two N_2_ IR components changes in the set of samples, indicating a dependence of Cu(i) site distribution on the composition. In particular, in the spectra obtained for the sample with Si/Al = 5 (first panel in Fig. 7a) the high frequency component (HF) around 2300 cm^–1^ seems dominant with respect to the low frequency one (∼2292 cm^–1^, LF). An opposite trend is observed for the sample with Si/Al = 29 (right hand panel), while the samples with Si/Al = 14 and 15 (middle panels) clearly show both components with relative intensity depending on the Cu/Al ratios. Spectral deconvolution was carried out on the high coverage bands reported in Fig. 7a (red curves), to semi-quantitatively estimate the abundance of the two Cu(i) sites. The procedure was first optimized for sample (Cu/Al = 0.1; Si/Al = 14), where the two components are more clearly defined. The resulting two best fits were then used as a starting point to analyse the other samples, keeping fixed the peak width and/or its shape (see the ESI Section 6 for details†). The average values of the normalized integrated areas calculated for each component are reported in the bar plot of Fig. 7b, while Table 2 also shows the corresponding average position and width. The bar plot in Fig. 7b also reports the total peak area, which will be discussed in the following.

**Table 2:** Average position, width and normalized integrated area of the two Cu(i)-N_2_ IR components (high frequency, HF and low frequency, LF) calculated by spectral deconvolution, including assignment

Composition (Cu/Al; Si/Al)	(0.1; 5)	(0.1; 14)	(0.5; 15)	(0.6; 29)
**HF ∼ 2300 cm** ^**–1**^ **ZCu(** **i** **) 6r**				
Position (cm^–1^)	2299.0 ± 0.2	2299.0 ± 0.8	2296.0 ± 0.1	2297.0 ± 1
Width (cm^–1^)	11.2 ± 0.2	11.9 ± 0.9	12.0 ± 0.2	12 ± 2
Norm. area (cm^–1^)	0.45	0.62	1.00	0.35

**LF ∼ 2292 cm** ^**–1**^ **ZCu(** **i** **) 8r**				
Position (cm^–1^)	2291 ± 1	2291.0 ± 0.3	2292.00 ± 0.01	2291.0 ± 0.2
Width (cm^–1^)	11 ± 3	7.9 ± 0.6	5.1 ± 0.1	6.7 ± 0.4
Norm. area (cm^–1^)	0.06	0.33	0.56	0.56

Concerning the quantitative estimations summarized in Fig. 7b and Table 2, which confirm the dependence of the concentration of the two components upon composition, some considerations have to be made. First, the extinction coefficients of the HF and LF bands can be different, since they depend on the specific interaction of the dinitrogen ligand with the Cu(i) sites. As discussed elsewhere,^84,86^ the formation of Cu(i)-N_2_ adducts involves both σ-donating and π-accepting interactions through the Cu(i) d orbitals and the dinitrogen filled 3σ_g_ and empty 1π_g_ molecular orbitals, respectively. These interactions weaken the N≡N bond, resulting in the observed redshift with respect to the (Raman active) gas-phase value of 2321 cm^–1^. Thus, a higher value for the redshift indicates a more effective σ-donation/d-back-donation interaction, which can be associated with a larger available space and/or a more effective cation ionic character.^84^ On this basis, we can assign the LF component at ∼2292 cm^–1^ to ZCu(i) sites in the 8r, and the HF one at ∼2300 cm^–1^ to ZCu(i) sites in the 6r, characterized by a more crowded coordinative environment, including two first-shell O_fw_ ligands at *ca.* 1.9 Å and two additional O′_fw_ atomic neighbours at *ca.* 2.6 Å (see DTF optimized structures in the ESI, Table S5†).

As pointed out in Section 2.4, these two ZCu(i) sites are expected to have very similar XANES signatures, and their possible co-existence can account for the increased structural disorder observed in correspondence to large ZCu(i) populations. These observations are in very good agreement with the FTIR results, since the sites are distinguishable only by using the weak N_2_ probe, while this is not the case with the CO probe (only one single band is observed in relation to Cu(i)-CO adducts at 2155 cm^–1^ on Cu/Al ∼ 0.44 and Si/Al ∼ 13 Cu-CHA,^86^ and on the other samples studied in this work).

Unfortunately, to the best of our knowledge, nothing can be said about the extinction coefficient of the two corresponding bands. For N_2_ adducts formed with alkali-metal ions, mainly characterized by an electrostatic interaction, the intensity of the IR vibration was found to be a function of the electric field, and thus proportional to the observed blue shift.^88^ Since the formation of Cu(i)-N_2_ adducts is driven by σ-donation/d-back-donation interactions, no direct relation between the observed redshift and the corresponding extinction coefficients can be drawn. On the other hand, the average peak width values reported in Table 2 show that the HF component is distinctly broader with respect to the LF one. The only exception is the sample with Si/Al = 5, where both the components are found with an equivalent peak width of *ca.* 11 cm^–1^. In this sample, however, the LF component represents a very minor contribution poorly defined in the deconvolution, as also evidenced by the higher error associated to its width. Such peak broadening further supports the assignment of the HF component to ZCu(i) in the 6r, where the N_2_ adduct could be perturbed by interactions with the framework in the small space of the 6r window. This broadening, however, could induce an overestimation of the relative amount of this site, based on the integrated peak areas (bar plot of Fig. 7b).

As mentioned above, Dědeček *et al.* showed a dependency on the intensity of the two Cu(i) luminescence bands on Cu loading.^87^ According to the authors, the 8r should be populated first (low Cu-loading) and reach saturation at Cu/Al ∼ 0.3, while the 6r is supposed to be populated only from Cu/Al = 0.1–0.2 and is not supposed to saturate at higher Cu/Al ratios (∼0.35). Unfortunately, the Si/Al ratio of the Cu-CHA zeolites studied by photoluminescence was not precisely stated, so that we cannot directly compare our results to those reported by Dědeček *et al.* However, these observations are in fair agreement with the estimated distribution of HF and LF Cu(i) sites on the two samples with Si/Al = 14 and 15 at low and high Cu loading (middle bars in Fig. 7b).

Finally, and more importantly in this context, the integrated area of the N_2_ peaks reported in Fig. 7a (highest coverage, red curves) is compared in Fig. 7b as coloured bars. These data (which are normalized with respect to Cu loading) can give an estimation about the Cu reducibility in the set of samples, similarly to what is discussed on the basis of XAS data. This estimation is limited by the following considerations: (i) the integrated area is not strictly proportional to the Cu(i) content, since the N_2_ adducts with the two possible ZCu(i) sites are likely to be characterized by different extinction coefficients, as discussed above; (ii) measurements were carried out at low temperature, while the XAS data was obtained *in situ* on the He-activated state at 400 °C; (iii) the efficiency of the self-reduction could be influenced by the activation conditions (vacuum *vs.* inert flow). Notwithstanding these limitations, the observed trend in reducibility among the set of samples is in good agreement with the XAS conclusions. Namely, reducibility increases passing from Si/Al = 5 to 15, where it reaches a maximum and then decreases at Si/Al = 29. Notice that, on the basis of what is discussed above about the broadening of the HF component, self-reducibility could be slightly overestimated in samples with a relatively high amount of the ZCu(i) 6r site, thus justifying the small discrepancies of these results with the XAS ones.

## 3 Conclusions

In this work, we have employed *in situ* XAS to monitor the He-activation process in a multi-composition platform of Cu-CHA zeolites, aiming to clarify the influence of the catalyst composition on the temperature-dependent Cu-speciation and reducibility. The abundance of redox-active Cu species is indeed expected to profoundly impact the performance of Cu-CHA in two of the currently most intensively studied processes over zeolite-based catalysts: NH_3_-SCR and direct methane to methanol conversion, both known to involve redox chemistry at the metal centres. After principal component analysis of the temperature-dependent multi-composition XANES dataset, we employed a multivariate curve resolution method to extract chemically-meaningful spectra and concentration profiles of pure components formed during He-activation as a function of the catalyst composition. Based on the spectroscopic fingerprints of each theoretical XANES and the correspondent temperature-dependent concentration profiles, we were able to reliably assign the theoretical spectra to pure Cu-species/sites. The assignment was subsequently corroborated by simulating the Cu K-edge XANES spectra for the correspondent model geometries. For the first time, this approach allowed us to rationalize in a quantitative frame the complex dynamics of Cu-cations in the CHA cages during the activation process. Of note, it will now be possible to use the theoretical XANES generated by MCR-ALS as references in the quantitative determination of Cu-speciation in Cu-CHA samples with arbitrary composition and/or treated under different conditions by simple linear combination analysis.

In summary, the formation of framework-interacting Cu-species from the mobile Cu(ii) aquo-complexes present at RT occurs, irrespectively of catalyst composition, *via* a Cu(ii) dehydration intermediate, peaking around 130 °C. Then, 1Al Z[Cu(ii)OH] and 2Al Z_2_Cu(ii) species develop almost simultaneously, with relative abundances strongly influenced by the Si/Al ratio in the parent zeolite. 1Al Z[Cu(ii)OH] species, dominant for samples with Si/Al in the 15–29 range, peak around 200 °C and then progressively decrease at elevated temperatures, in favour of reduced 1Al ZCu(i) species. Conversely, 2Al Z_2_Cu(ii) sites, dominant at Si/Al = 5, reach a steady population in the 200–300 °C range and remain stable until 400 °C. Cu-speciation at 400 °C can be described for all samples as a combination of redox-active 1Al sites, in their oxidized and reduced form, and redox-inert 2Al Z_2_Cu(ii) sites.

By quantitatively monitoring the composition-driven Cu-speciation during He-activation of Cu-CHA, we highlighted novel traits of complexity accompanying the transition from mobile Cu-complexes to framework-interacting Cu-species and the subsequent self-reduction process. In particular, the high reducibility observed in the low-loading sample (Cu/Al = 0.1) with Si/Al = 14 and the almost simultaneous development of 1Al and 2Al populations both contrast with an ideal 2Al site saturation scenario, often assumed in the current literature. Moreover, the reducibility level of the 1Al sites is shown to depend on Si/Al (optimal reducibility for Si/Al = 15, lower reducibility at Si/Al = 19 and 29), evidencing that self-reduction proceeds though a cooperative multi-step process involving proximal acid sites, whose availability is ultimately determined by Al density and distribution in the zeolite.

The MCR-ALS XANES results are also combined with multi-component fits of the *in situ* EXAFS spectra collected at 400 °C after He-activation, to independently validate the employed reconstruction method and access detailed structural information on the preferred Cu local coordination environment associated with 1Al and 2Al sites. EXAFS fits substantially confirm the Cu-speciation evaluated from MCR-ALS analysis of temperature-dependent *in situ* XANES, but evidence that self-reduction is accompanied by higher levels of structural disorder in the Cu local environment. The composition-driven reducibility trends and the nature of the ZCu(i) structural component are further investigated using FTIR spectroscopy. Low-temperature IR spectra of N_2_ adsorbed on vacuum-activated Cu-CHA catalysts with different compositions clearly show two components at 2292 cm^–1^ and at 2300 cm^–1^, that we rationalize in terms of the different cation local environment for 1Al ZCu(i) sites in the 8r and 6r, respectively.

All of these findings synergically demonstrate that reducibility in Cu-CHA results from the balance between two distinct Cu-speciation-related effects: (i) at low Si/Al values (*e.g.* Si/Al = 5), redox-inert 2Al sites in the 6r are favoured; (ii) at high Si/Al values (*e.g.* Si/Al = 29), redox-active 1Al sites largely dominate, but the self-reduction of the correspondent Z[Cu(ii)OH] complexes is hindered due to the low interaction probability among proximal acid sites, yielding a counterintuitive enhancement of the relative Cu(ii) fraction in the He-activated state.

Finally, this study addresses the grand challenge of characterization in heterogeneous catalysis, demonstrating the potential of chemometric techniques, such as MCR-ALS, in the modelling of a complex spectroscopic dataset, and enabling an unprecedented level of understanding in a complex multi-component system.
