# Supplementary material for: Composition-driven Cu-speciation and reducibility in Cu-CHA zeolite catalysts: a multivariate XAS/FTIR approach to complexity
Source: Chem Sci. 2017 Jul 24;8(10):6836–51. doi: 10.1039/c7sc02266b (PMC5644121; doi:10.1039/c7sc02266b)
Supplement: Supplementary file 1 [file SC-008-C7SC02266B-s001.pdf]

This journal is © The Royal Society of Chemistry 2017

Supporting information

Composition-driven Cu-speciation and reducibility in Cu-CHA zeolite catalysts: a multivariate XAS/FTIR approach to complexity

A. Martini[a], E. Borfecchia[a,b]*, K. A. Lomachenko[c,d], I. A. Pankin[a,d], C. Negri[a], G. Berlier[a], P. Beato[b], H. Falsig[b], S. Bordiga[a], C. Lamberti[d,e]

[a]*Department of Chemistry, NIS Centre and INSTM Reference Center, University of Turin, Via Giuria 7, Turin, 10125 Italy.*
[b]*Haldor Topsøe A/S, Haldor Topsøes Allé 1, Kgs. Lyngby, 2800 Denmark.*
[c] *European Synchrotron Radiation Facility, 71 avenue des Martyrs, CS 40220, Grenoble Cedex 9, 38043 France.*
[d]*IRC "Smart Materials", Southern Federal University, Zorge str. 5, Rostov-on-Don, 344090 Russia.*
[e]*Department of Chemistry, CrisDi Centre and INSTM Reference Center, University of Turin, Via Giuria 7, Turin, 10125 Italy.*

Contents

1 Materials and Methods 2
1.1 Samples description and synthesis details 2
1.2 In situ XAS 2
1.2.1 *In situ* gas flow setup 2
1.2.2 *In situ* XAS data collection and data reduction procedures 2
1.3 In situ FTIR spectroscopy 3
1.4 MCR-ALS: details on the method and its implementation 3
1.4.1 General description of the MCR-ALS method 3
1.4.2 Details on the MCR-ALS application in the present study 4
1.5 DFT-based XANES simulation 5
2 Determination of N_{pure} by principal component analysis (PCA) 5
2.1 Hypothesis of negligible temperature-dependence in the XANES region 6
3 MCR-ALS results for different values of N_{pure} 9
3.1 Reconstruction results with downsized (N_{pure} = 3, 4) or upsized (N_{pure} = 6) PC space 9
3.2 R-factor analysis for MCR-ALS reconstructions in downsized or upsized PC spaces 13
4 Assignment of MCR-ALS $\mu^{pure}(E)$ curves for PC1 and PC5: additional information 16
4.1 Comparison with experimental references 16
4.2 XANES simulations for $[Cu(II)(H_2O)_5]^{2+}$ and $[Cu(II)(H_2O)_4]^{2+}$ 16
5 Details on EXAFS analysis 17
5.1 EXAFS spectra in k-space 17
5.2 Fit details and shells model adopted for EXAFS analysis 17
5.3 Results of single-component fits on the 'purest' He-activated states 18
5.4 Details on multi-component EXAFS fits on the whole multi-composition dataset 21
5.5 EXAFS fitting results in k-space 21
6 Details on IR spectral deconvolution 22
7 References 24

1 Materials and Methods

1.1 *Samples description and synthesis details*

The CHA zeolites with Si/Al ~ 14, 15, 19, 29 were prepared as reported earlier,[1] modulating the composition of the synthesis gel to obtain the targeted Si/Al ratios. Copper ions were introduced by suspending the calcined CHA zeolite in copper(II) acetate solutions with concentration tuned as a function of the targeted Cu/Al and stirred at room temperature for 24 h.

CHA zeolite with Si/Al ~ 5 was synthesized with slight modification of the recipe described by Pham *et al.*[2] Briefly, 32.9 g de-ionized water was mixed with 13.4g N,N,N-trimethyl-1-adamantanamine hydroxide (TMAdaOH, 25 wt %, Sachem Inc.) and 0.2 g sodium hydroxide (Sigma-Aldrich, > 98.0%). To the resulting solution, 20 g of sodium silicate solution (26.5 wt % SiO_2, Sigma Aldrich) and 2 g of USY (CBV 500) were added respectively. The final mixture was transferred into a Teflon lined autoclave and crystallized for 6 days in a pre-heated oven (140 °C) with an inset that tumbles the autoclave (25 rpm). The product was then recovered by filtration, washed with de-ionized water and dried at 105 °C overnight. The organic temple was removed from the zeolite by calcination at 580 °C. The calcined product was ion-exchanged (3 × 2 hours at 80 °C) to ammonium from using 1 M ammonium nitrate solution, followed by calcination at 500 °C to desorb ammonia and from proton form of the zeolite.

Finally, for copper exchange, the required amount of copper(II) acetate monohydrate (Sigma-Aldrich, 99.99%) was dissolved in water (150 g water per 1 gram of zeolite), and the proton form of the zeolite was added to the solution. The resulting mixture was stirred overnight at room temperature; the copper exchanged zeolite was recovered by filtration and dried at 50 °C.

The composition of the investigated samples, evaluated from ICP-OES elemental analysis, is reported in Table S1.

Table S1. Summary of compositional properties of the investigated Cu-CHA samples.

Sample name (Cu/Al; Si/Al)	% wt Cu	Cu/Al	%wt Al	%wt Si	Si/Al
**(0.1; 5)**	1.51	0.11	6.03	31.0	4.94
**(0.3; 5)**	4.20	0.33	5.42	27.6	4.89
**(0.1; 14)**	0.76	0.13	2.47	36.1	14.06
**(0.5; 15)**	2.64	0.47	2.38	37.5	15.12
**(0.6; 19)**	2.59	0.56	1.98	38.5	18.68
**(0.6; 29)**	1.71	0.56	1.30	39.5	29.17

1.2 *In situ XAS*

1.2.1 *In situ* gas flow setup

In situ XAS data were collected at the BM23 beamline of the European Synchrotron Radiation Facility (ESRF, Grenoble, France). To measure the Cu-CHA catalysts under controlled conditions in terms of temperature (T) and gaseous atmosphere during the dehydration process, we employed the Microtomo reactor cell[3] designed by the ESRF Sample Environment Group integrated in a devoted gas-flow setup, as described in detail in our previous works.[4, 5] *In situ* XAS experiments during He-activation were performed heating the samples from room temperature (RT) to 400 °C, with an heating rate of 5 °C/min and flowing in the Microtomo reactor cell 100 ml/min of He (quality 5.7). A moisture trap was employed to further prevent any unwanted water contamination in the He gas stream arriving to the cell inlet.

1.2.2 *In situ* XAS data collection and data reduction procedures

In situ Cu K-edge XAS data were collected in transmission mode, using double-crystal Si (111) monochromator for the incident energy scan and ionization chambers for the detection of incident and transmitted photons. The chambers were filled with a He/Ar mixture up to 2.2 bar with the partial pressure of

argon of 0.1 and 0.3 bar for I_0 and I_1 chambers, respectively. For an accurate edge energy calibration, a copper foil was measured simultaneously with all the acquired spectra using a third ionization chamber I_2.[6, 7]
The Cu-CHA catalysts were measured in the form of a self-supporting wafers (optimized samples weights in the 90−140 mg range for 1.3 cm^2 area pellets, resulting in edge jumps $\Delta\mu x$ in the 0.3−1.1 range for a total absorption after the edge of $\mu x = 2.5$) fixed inside the Microtomo reactor cell.
Two different XAS acquisition modes were employed. The evolution of the XANES features during He-activation was monitored with faster acquisitions of ~ 6 min, from 8900 to 9182 eV (up to ~ 7 $Å^{-1}$), allowing to appreciate the variation of the XAS signal as a function of the temperature (pre-edge region energy step = 5 eV, edge region energy step = 0.3 eV; EXAFS part collected with variable sampling step in energy, resulting in $\Delta k = 0.08$ $Å^{-1}$; integration time was 1 s/point in all regions). The resulting time/temperature-dependent *in situ* XANES datasets for Cu-CHA at different compositions were subjected to multi-way Multivariate Curve Resolution (MCR) analysis as described below (see Section 1.4). All the He-activated catalysts, after stabilization of the XANES features at 400 °C in He-flow, were further characterized by two consecutive higher-quality XAS scans of ~ 30 min each, collected from 8800 to 9955 eV (up to ~ 16 $Å^{-1}$) with enhanced k-space sampling ($\Delta k = 0.035$ $Å^{-1}$) and acquisition time in the EXAFS region, quadratically increasing with k from 1 to 4 s/point to account for the lower signal-to-noise ratio as k increases. The final spectra employed for EXAFS fitting were obtained as the average of the two $\mu(E)$ curves corresponding to the consecutive scans, after checking reproducibility among the two acquisitions.
All the collected XAS spectra were aligned in energy using the corresponding Cu metal foil spectra detected by the I_2 ionization chamber and normalized to unity edge jump using the Athena software from the Demeter package.[8] The extraction of the $\chi(k)$ function was also performed using Athena program[8], and R-space EXAFS spectra were obtained by calculating the Fourier transform of the $k^2\chi(k)$ functions in the (2.4 – 12.4) $Å^{-1}$ k-range.

1.3 In situ FTIR spectroscopy

For FTIR measurements, thin self-supporting wafers of each Cu-zeolite were prepared and placed inside an IR cell designed to allow *in situ* high-temperature treatments, gas dosage, and low-temperature measurements. Prior to adsorption experiments, samples were pre-treated under dynamic high vacuum conditions ($p < 10^{-4}$ mbar) at 400 °C for 2 h. The IR spectra were recorded at 2 cm^{-1} resolution on a Bruker 66 FTIR spectrometer equipped with a deuterium telluride gallium sulfide (DTGS) detector; 64 interferograms were averaged for each spectrum. The spectrum measured before N_2 dosage was used as reference to obtain the reported background-subtracted spectra. Adsorption experiments were performed at low temperature, cooling the cell with liquid nitrogen; the actual sample temperature (under the IR beam) was estimated around −160 °C. The absorbance of the zeolite overtone modes in the 1750–2100 cm^{-1} region was used for normalization, to account for differences in pellet thickness. Moreover, each set of spectra was further normalized by dividing for the Cu wt% of the specific investigated catalyst (see Table S1), in order to have a set of data comparable to XAS data, which are intrinsically normalized with respect to Cu content.

1.4 MCR-ALS: details on the method and its implementation

1.4.1 General description of the MCR-ALS method

Multivariate Curve Resolution - Alternating Least Squares (MCR-ALS) is a powerful chemometric algorithm that permits to decompose an experimental mixture of spectra $\mathbf{D}$ into pure contributions, consisting of concentration profiles $\mathbf{C}$ and the corresponding spectra $\mathbf{S}$ of different chemical compounds (i.e. bilinear decomposition):

$$\mathbf{D} = \mathbf{C} \cdot \mathbf{S}^{\mathbf{t}} \tag{1}$$

where $\mathbf{S}^{\mathbf{t}}$ denotes the transpose of matrix $\mathbf{S}$.
The first step of the algorithm consists in the determination of the number of components that characterize the entire dataset (referred to as N_{pure} in the following). To this purpose, Principal Component Analysis (PCA) can be performed. This method aims to describe the maximum variance of the dataset. It is worth underlying that the number of pure spectra of the mixture equals the number of principal components.[9, 10]

In this context, the examination of the magnitude of singular values (i.e. the covariance matrix eigenvalues related to **D**) is typically used. Indeed, singular values related to effective chemical contributions are large, whereas singular values related to noise are relatively small and similar one among the others.[9, 10]
The initial estimates in MCR-ALS can be either concentration profiles or spectra of pure components. Their generation requires initial guesses with the same general properties of the profiles that has to be recovered. Initial random values are generally not helpful, because the expected profiles do not have such a numerical structure.[9] Efficient methods employed to generate initial estimates for any kind of datasets include the so-called *purest variable selection* methods, such as SIMPLISMA.[9, 11] SIMPLISMA proceeds by selecting the most dissimilar rows or columns in the original dataset **D** and providing, as a consequence, initial estimates of spectra or concentration profiles, respectively. However, it is generally incorrect to link the profiles extracted by these methods with pure component profiles, because selectivity cannot be always ensured.[9]
Once initial estimates are obtained, optimization takes place until convergence is achieved. The general minimization step in the iterative optimization can be expressed as follows:[9]

$$\min\|\mathbf{D} - \mathbf{C} \cdot \mathbf{S}^{\mathbf{t}}\| \qquad (2)$$

taking into account that $\mathbf{C}$ and $\mathbf{S}$ matrices are calculated in each iterative cycle.
In order to control the convergence, figures of merit related to the model fit are used, such as the lack of fit (% LOF):[9, 12]

$$\%\mathrm{LOF} = \sqrt{\frac{\sum_{i,j} e_{ij}^2}{\sum_{i,j} d_{ij}^2}} \qquad (3)$$

where e_{ij} and d_{ij} are the elements (i, j) of the residual matrix **R** and original dataset **D**, respectively.
The optimization usually finishes when the difference of the model fit between two consecutive iterations does not improve significantly (e.g. a difference of less than 0.1% among the LOF between two consecutive iterations is achieved). However, the MCR-ALS algorithm is affected by the so-called ambiguity phenomenon.[9, 12] This means that different combinations of concentration profiles and spectra can describe equally well, in term of model fit, the original dataset.[9] In order to suppress the ambiguity related to the algorithm solutions and obtain physically/chemically meaningful concentration profiles and pure spectra, it is necessary to introduce a set of constraints. For this reason, in each iterative cycle, the computed least squares profiles (spectra, concentrations or both) are modified so that they obey to the conditions imposed by preselected constraints such as *non negativity*, *unimodality*, and *closure*.[9, 12]

1.4.2 Details on the MCR-ALS application in the present study

In our study, we employed the GUI developed by Jaumot and co-workers[12] (freely downloadable at http://www.mcrals.info/), analysing simultaneously in a 'multi-way' reconstruction the whole multi-composition dataset (13 temperature-dependent XANES spectra for each catalyst composition, 6 investigated compositions, for a total of 78 spectra). The experimental spectra were analysed in the (8975−9020) eV energy range. The ALS routine was run with initial concentration/spectra guessed using the purest variable detection method (SIMPLISMA algorithm[11]) and using only soft constrains: non-negativity for both pure spectra and concentration profiles, and closure to 1 for concentration profiles, $\sum_{\mathrm{i=1}}^{\mathrm{N_{pure}}} w_{\mathrm{i}}^{\mathrm{pure}}\left(\frac{Cu}{Al};\frac{Si}{Al};\,T\right) = 1$, ∀T), since we are working with an element selective technique, which probes all the present Cu-species, averaging their response with temperature/composition-dependent relative abundance of each Cu-species, namely $w_{\mathrm{i}}^{\mathrm{pure}}\left(\frac{Cu}{Al};\frac{Si}{Al};\,T\right)$, as the weight. In correspondence of the optimal value of N_{pure} (determined as described below in Section 1.5), the MCR-ALS routine converged successfully (i.e %LOF< 0.1) after 21 interactions. The values of a series of quality indicators for the achieved reconstruction of the experimental dataset are reported in Table S2.

Table S2. Quality indicators of the MCR-ALS analysis (N_{pure} = 5) of the global *in situ* XANES dataset reported in Figure 1a in the main text.

MCR-ALS Quality Indicator	Value
Std. deviation of residual vs exp. Data	0.0083
Fitting error (LOF) in (%) of PCA	0.6989
Fitting error (LOF) in (%) of exp.	0.8878
Percentage of variance explained at the optimum	99.9921

1.5 DFT-based XANES simulations

Structures used for EXAFS fitting and XANES calculations were obtained by means of DFT geometry optimization. For framework-interacting Cu-species (namely *1Al* Z[Cu(II)(OH)], *1Al* ZCu(I), and *2Al* Z_2Cu(II) sites, see Figure 5a in the main text) we employed the models reported in our previous work,[5] to which the reader is referred for additional computational details. $[Cu(H_2O)_n]^{2+}$ models were optimized independently, since these species are known to be localized in the large cavities of CHA structure without direct coordination to the zeolitic framework. Geometry optimization for all Cu-species/sites was carried out with the ADF2012 software, [13, 14] using Slater-type TZ2P basis sets for all atoms together with the frozen core approximation (up to 2p frozen for Cu, Si and Al, 1s frozen for O).[15] The PBE functional was chosen to account for exchange correlation effects,[16] and relativistic effects were considered by means of the scalar ZORA approximation.[17] Convergence criteria were set to 0.001 Hartree, 0.001 Hartree/Å and 0.01 Å for energy, gradients and atomic displacements, respectively. Cu K-edge XANES spectra were simulated using the molecular orbitals calculated by ADF. After geometry optimization, an all-electron single point calculation was performed for each structure using the very large QZ4P basis set in order to obtain high-quality data for both core and valence orbitals. The resulting molecular orbitals were subsequently projected onto the cubic grid centered around the absorbing atom. Grid dimensions were 1 Å in each direction, comprising 51^3=132651 points. The XANES spectra were obtained by numerical volume integration of the 1s core orbitals with unoccupied valence orbitals within the dipole approximation. Integration was performed by an in-house software developed by Dr. G. Smolentsev.[18]. At the final stage, energy-dependent Lorentzian broadening of the obtained transitions was performed to match the experimental resolution. Resulting spectra were aligned along the energy axis according to the energy of Cu 1s orbital in corresponding models, and then a rigid shift of all the simulated spectra was performed to compare them with the $\mu_i^{pure}(E)$ curves obtained from MCR-ALS analysis.

2 Determination of N_{pure} by principal component analysis (PCA)

The first step in the analysis of the *in situ* XANES dataset collected during He-activation of Cu-CHA involved the determination of the number of pure Cu-species to combine in order to explain the variance observed in the experimental spectral series above the noise level. To this aim, we performed principal component analysis (PCA) of *in situ* XANES series (He-activation from 25 to 400 °C) for each of the six investigated samples.

As a first approach to identify the best number of principal components (PCs) to include in the global analysis of the T-dependent multi-composition XANES dataset, we compared the 'Scree plots' (PC eigenvalues as a function of PC number, see Figure S1a) for the whole sample series. Although in several cases a rather smooth scree plot is obtained, a first global 'knee' can be located at the 5th PC. A further increase in the number of PCs yield modest variations in the eigenvalues (please note that a logarithmic scale is used for the ordinate axis in Figure S1a), and after the 8th PC we observe full stabilization of the eigenvalues for the whole sample series.

Albeit informative, the Scree plots can only yield qualitative indications. Hence, we evaluated the number of significant PCs, N_{pure}, in the T-dependent XANES series for each sample using two additional quantitative approaches, i.e. the Malinowski F-test (5% significance level) and the IND-factor.[10] As reported in Table S3, N_{pure}(F-test) selected for the different samples range from 4 to 6. As expected, for each sample, N_{pure}(IND) ≥ N_{pure}(F-test), with values comprised in the 4−7 range depending on the composition.

Aiming at a global analysis of the multi-composition T-dependent dataset, we calculated the average number of significant PCs $\langle N_{pure} \rangle$ evaluated with the two approaches for the whole sample series (see Table S3), obtaining $\langle N_{pure} \rangle = 5 \pm 1$ and $\langle N_{pure} \rangle = 6 \pm 1$ using Malinowski F-test (5% significance level) and the IND-factor, respectively (errors given by standard deviation $\sigma_{\langle N pure \rangle}$).

Moreover, since the IND factor suggests that in all analysed catalysts one (or two) additional components could be present compared to the F-test predictions (see Table S3), we also examined the abstract component matrix to assess the significance of these additional factors.[10, 19] Figure S1b reports the first six abstract components for a typical Cu-CHA sample, composition: (Cu/Al = 0.6; Si/Al=19). It can be noted that the first five factors represent most of the signal while the sixth is mainly dominated by noise. Equivalent results have been obtained for other investigated Cu-CHA samples. Based on these phenomenological evidences and on

the qualitative indications from Scree plots (see Figure S1a), we selected $\langle N_{pure} \rangle = 5$ for the subsequent analysis.

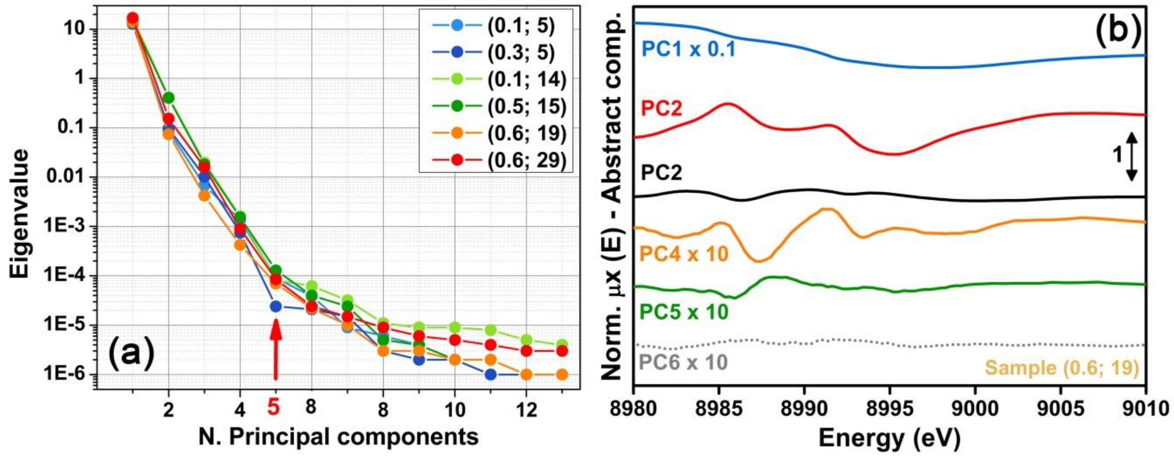

Figure S1. (a) Scree plots obtained after PCA of the *in situ* XANES datasets (He-activation from 25 °C to 400 °C, 13 spectra per sample) for Cu-CHA at different composition. (b) First six abstract components determined by PCA analysis on the *in situ* XANES dataset collected for a typical Cu-CHA sample, composition: (Cu/Al = 0.6; Si/Al=19), during He-activation from RT to 400 °C.

Table S3. Evaluation of the number of significant PCs in *in situ* XANES datasets (He-activation from 25 °C to 400 °C, 13 spectra per sample) for Cu-CHA at different composition using the Malinowski F-test (significance level α=5%) and the IND factor.

Cu-CHA samples (Cu/Al; Si/Al)	N_{pure}	
	Malinowski F-test (α=5%)	IND-factor
**(0.1; 5)**	6	6
**(0.3; 5)**	4	4
**(0.1; 14)**	4	6
**(0.5; 15)**	5	7
**(0.6; 19)**	5	6
**(0.6; 29)**	5	5
$\langle N_{pure.} \rangle \pm \sigma_{\langle Npure \rangle}$	5 ± 1	6 ± 1

2.1 *Hypothesis of negligible temperature-dependence in the XANES region*

It is important to note that PCA of the temperature-dependent XANES dataset described above has been performed assuming that any systematic thermal dependence of the XANES signal is negligible with respect to the Cu-speciation-related contributions to the variance observed in the experimental dataset. Practically, this assumption translates, after MCR-ALS, in the possibility to decouple the data matrix in temperature-independent pure species XANES spectra $\mu_i^{pure}(E)$ and temperature-dependent pure species concentration profiles $w_i^{pure}(T)$

The impact of thermal disorder is well known to significantly affect the XAS signal in the EXAFS region, leading to a significant damping of the fine structure. After the absorption edge, the damping can be modelled using a Debye-Waller factor σ^2, through the expression $\exp(-2\sigma^2 k^2)$, where σ^2 represents the correlated mean-square radial vibrational amplitude of the bond to neighbouring atoms.[20, 21] To estimate the impact of such a thermally-induced damping in the different energy-ranges of interest in our study, we considered typical σ^2 values for first-shell Cu–O bonds in vacuum/He-activated Cu-zeolites at RT and at the highest temperature considered in the present study, i.e. 400 °C. The value of σ^2 (T = 400 °C) = 0.007 Å^2 has been directly derived from the EXAFS analysis reported in the present work (see Section 2.4 in the main text) while, for an indicative

RT value, we refer to a previous study on vacuum-activated Cu-ZSM-5 cooled in vacuum and measured at 27 °C, reporting σ^2 (T = 27 °C) = 0.005 Å^2.[22]
Figure S2 shows the exp($-2\sigma^2 k^2$) damping factor as a function of k calculated for the typical σ^2 values indicated above at RT (blue curve) and 400 °C (red curve). The vertical dashed bars indicate the k-space ranges of interest for XANES analysis (up to 9020 eV, 19 eV above the absorption edge E_0 = 8991 eV, corresponding to ~ 2.6 Å^{-1}) and EXAFS fit (2.4-11.0 Å^{-1}). Within the XANES region, the energy positions of the highly informative white-line region (WL, k ~ 1.1 Å^{-1}) and of the last XANES feature (LF, k ~ 2.0 Å^{-1}) observed in the experimental dataset before the flat post-edge region are also indicated as light-grey dotted vertical bars.
From Figure S2 it is evident that the damping effect in the k-space range of interest for XANES PCA and subsequent MCR-ALS analysis is weak, and smoothly varying with temperature in the experimentally relevant range. In particular, the difference in the exp ($-2k^2\sigma^2$) damping factor (grey curve in Figure S2, calculated as $\Delta(k) = \exp[-2k^2\sigma^2(RT)] - \exp[-2k^2\sigma^2(400)]$), in the 'worst' case, at the end of the region used for MCR-ALS analysis of XANES spectra (k ~ 2.6 Å^{-1}) is ~ 2.5 x 10^{-2}. However, the last significant XANES feature occurs at ~ 9006 eV (k ~ 2.0 Å^{-1}): here the difference in the damping factor is already significantly lower, Δ ~ 1.5 x 10^{-2}. In addition, at the white-line position (that is the region where the activation process induces the largest changes in the experimental XANES spectra), occurring at ~ 8996 eV (k ~ 1.1 Å^{-1}), the difference in the damping factor is Δ ~ 4.8 x 10^{-3}.

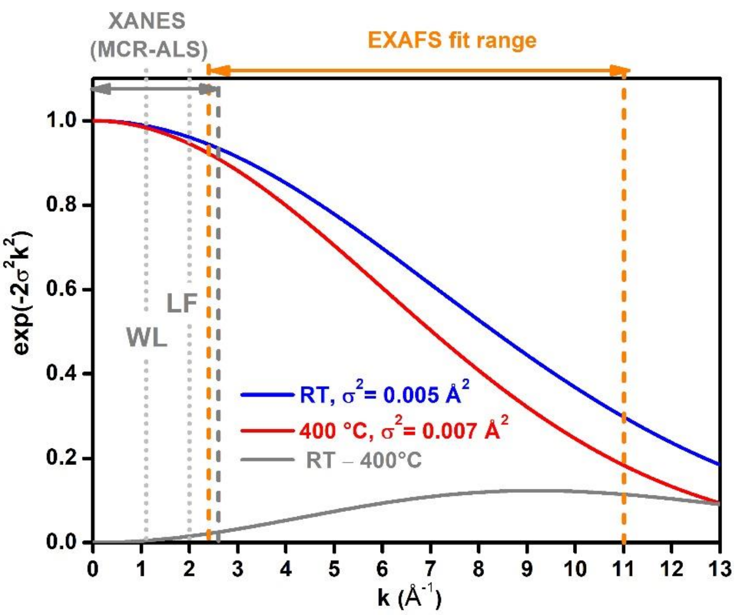

Figure S2. Behavior of the exp($-2\sigma^2 k^2$) damping factor as a function of k calculated using typical σ^2 values for first-shell Cu–O bonds in vacuum/He-activated Cu-zeolites at RT, σ^2 (T = 27 °C) = 0.005 Å^2, from ref.[22], and at 400 °C, σ^2 (T = 400 °C) = 0.007 Å^2, from the EXAFS analysis reported in the present work (see Section 2.4 in the main text). The k-dependent variation $\Delta(k)$ of the damping factor from RT to 400 °C is also reported in grey. The vertical dashed bars indicate the k-space ranges of interest for XANES analysis (grey bars: up to 19 eV above the absorption edge E_0 = 8991 eV, corresponding to 2.6 Å^{-1} and EXAFS fit (orange bars, 2.4-11.0 Å^{-1}). Within the XANES region, the energy positions of the highly informative white line region (WL) and of the last XANES feature observed in the experimental dataset before the flat post-edge region (LF) are also indicated as light-grey dotted vertical bars.

In order to better estimate the thermal effects on our factor analysis over the whole used k-range, we corrected the five relevant abstract components (PC1-PC5) and the first neglected one (PC6) by the k-dependent damping factor at the initial (RT) and final (400 °C) temperatures. These corrected components are reported in Figure S3 for a representative sample (composition: (0.6; 19)) as blue and red curves, respectively. In our factor analysis, we neglected the effect of temperature; consequently, the actual abstract components used (also reported in Figure S1b) lie in between the red and the blue ones reported in Figure S3. Figure S3 reports also the differences (400°C - RT) between the temperature-corrected components, as grey curves, right ordinate axis.

For all components, in the whole k-range relevant for XANES analysis, the absolute value of the error resulting from the temperature-independence approximation (grey curves) is ca. two orders of magnitude smaller than the amplitude of the corresponding abstract components. Moreover, this ratio is even smaller in the shorter k-range which contains the most informative XANES features (up to 2.0 Å^{-1}); in the subsequent 2.0-2.6 Å^{-1} range, all the abstract components are much flatter, and consequently less informative.

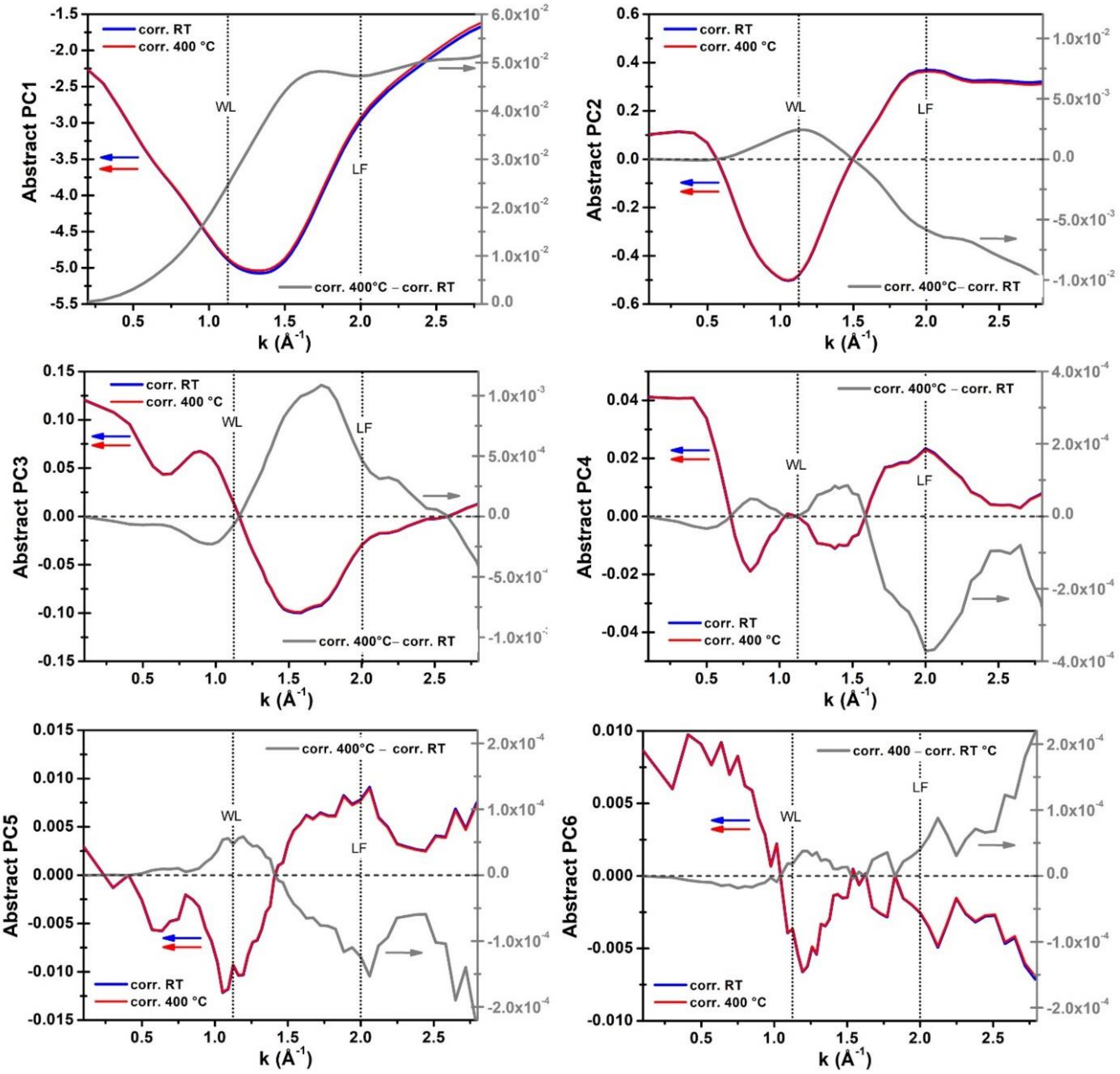

Figure S3. Left ordinate axis: first five significant abstract components (PC1-PC5) and first excluded one (PC6) corrected by the k-dependent damping factor at the starting (RT, blue curves) and final (400 °C, red curves) temperatures for a representative sample, composition: (0.6; 19). Right ordinate axes: k-dependent differences between the temperature-corrected components (400°C - RT), shown as grey curves.

Based on this evidences and in line with several previous studies applying PCA analysis to temperature-dependent XANES data series[19, 23-26] we can thus safely work under the hypothesis of temperature-independence of the XANES signal, whereas such assumption could not be applied to PCA analysis of temperature dependent EXAFS data, as evidenced in the work by Cassinelli *et al.*[26]

It is finally worth to note that the considerations reported here above cannot be rigorously applied in the XANES energy range before the absorption edge (8975-8991 eV in the present study) and in its closest proximity. Here, different approaches have been proposed to model the temperature dependence of the XANES in the pre-edge region and edge-position,[27-30] with a general conclusion that such effects have a rather limited influence in the absence of specific temperature-induced structural transitions.

3 MCR-ALS results for different values of N_{pure}

3.1 Reconstruction results with downsized (N_{pure} = 3, 4) or upsized (N_{pure} = 6) PC space

To corroborate the selection of $N_{pure} = 5$ as the optimal number of pure Cu-species contributing in the experimental multi-composition *in situ* XANES dataset, we repeated the MCR-ALS protocol in correspondence of downsized and upsized PC spaces around the optimal dimension identified by statistical analysis, as described in Section 1.5. From qualitative analysis (see Section 2.1 in the main text), is it clear that the minimum PC space dimension able to account for the key modifications in our spectral series is obtained for $N_{pure} = 3$. This basic set of Cu-species should include (i) an initial Cu(II) species characterized by an intense white line peak and a flat edge-rising region and two 'dehydrated' Cu species formed upon thermal treatment: (ii) a Cu(II) species with a lower white-line intensity and a well-defined edge-rising shoulder in the 8985−8987 eV range, and (iii) a Cu(I) species to account for the prominent edge-rising peaks developing from 8982 eV upwards and the erosion of the 1s → 3d pre-edge peak in the most reducible samples. Hence, to explore the effect of a downsized PC space on the MCR-ALS reconstruction, we used N_{pure} = 3, 4, while keeping exactly the same parameters and constrains as for the optimal $N_{pure} = 5$ case (see Section 1.4.2). Equivalently, we tested the effect of an additional PC beyond the optimum, running the MCR-ALS analysis also for $N_{pure} = 6$.

Figure S4 compares the $\mu_i^{pure}(E)$ spectra obtained in correspondence of downsized, part (a), and upsized, part (b), PC spaces with the theoretical XANES retrieved for optimum PC space dimension $N_{pure} = 5$, reported and discussed in details in the main text, Section 2.3.

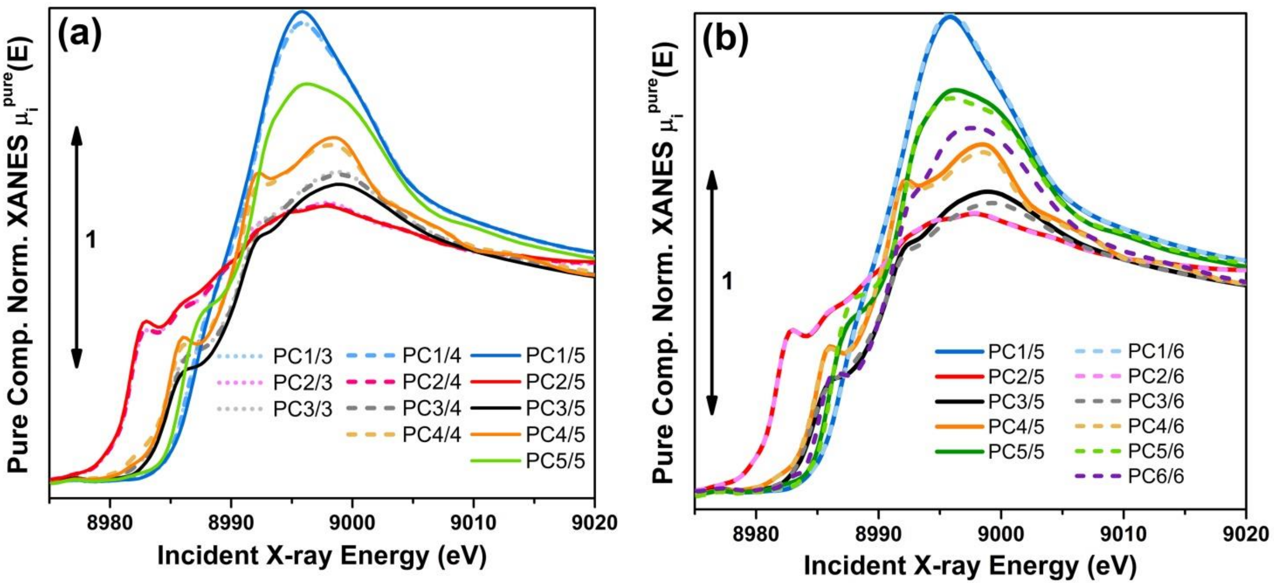

Figure S4. (a) XANES spectra of pure components $\mu_i^{pure}(E)$ derived from MCR-ALS using downsized PC space (N_{pure} = 3, 4) compared with the correspondent theoretical $\mu_i^{pure}(E)$ curves obtained for optimal value of $N_{pure} = 5$. (b) The same as (a), but comparing the MCR-ALS results for upsized ($N_{pure} = 6$) and optimal ($N_{pure} = 5$) PC spaces.

Noteworthy, variations of N_{pure} in the 3−6 range do not result in strong perturbations of the pure spectra, so that while incrementing the number of components, the previously modelled $\mu_i^{pure}(E)$ curves are basically unchanged. This confirms the overall stability of the reconstruction, and the robustness of the initial guesses obtained by using the *purest variable selection* method.

We observe how in the case of $N_{pure} = 3$, a dehydrated Cu(II) spectrum almost equivalent to the one assigned to tridentate *1Al* Z[Cu(II)OH] complexes in the 5-PC model is obtained (PC3/3 in Figure S4a), whereas the characteristic XANES features emerging at Si/Al = 5 (well-resolved peaks at ~ 8998 and ~ 8992 eV, and a broad post-edge peak in the 9005−9010 eV range) are not modelled. To account for such features, an additional dehydrated Cu(II) species is clearly required, which indeed readily emerges when the reconstruction is performed with $N_{pure} = 4$ (PC4/4 in Figure S4a), in relation with *2Al* $Z_2Cu(II)$ species in the *6r*. Comparing the results for $N_{pure} = 4$ and $N_{pure} = 5$, we observe only very minor changes in all the $\mu_i^{pure}(E)$ curves. Importantly the additional $\mu_5^{pure}(E)$ in the 5-PC model shows a distinct rising-edge shoulder at ca. 8987 eV, which is not present in any of the other theoretical XANES. In contrast, the $N_{pure} = 6$ model yield a $\mu_6^{pure}(E)$ spectrum which

is rather well described as an average between the $\mu_3^{pure}(E)$ and $\mu_5^{pure}(E)$ curves retrieved in the optimal 5-PC model (PC6/6 in Figure S4b).

Further insights are obtained by comparing the concentration profiles derived from MCR-ALS analysis in correspondence of different values of N_{pure}. The results for downsized (N_{pure} = 3, 4) and upsized (N_{pure} = 6) PC spaces are compared with the N_{pure} = 5 case in Figure S5 and Figure S6, respectively, for each of the investigated catalyst compositions. Here, in general, we observe significant readjustments of the concentration profiles when the dimension of the PC space is varied around the optimum.

Passing from N_{pure} = 3 to N_{pure} = 4, as expected, we observe major modifications in the concentration profiles for Si/Al = 5 catalysts, where the newly added PC4/4 dominates in the high-T region to the expenses of PC3/4. Consistently with the minor contribution of Z_2Cu(II) species at higher Si/Al values, the transition from 3- to 4-PC models has a minor impact on the concentration profiles for Si/Al = 14, 15, and is almost negligible at Si/Al = 19, 29.

The effect of the addition of PC5 significantly re-modulates the concentration profiles for all probed compositions, affecting in particular the populations of hydrated Cu(II) (PC1) and Z[Cu(II)OH] species (PC3), whereas the profiles for ZCu(I) (PC2) and Z_2Cu(II) (PC4) are perturbed only to a minor extent. The profiles retrieved for PC5 in the 5-PC model are similar for all the investigated compositions: a concentration peak is always observed at the intersection between the T-decreasing profile of PC1 and the T-increasing one for PC3. This behaviour is well explained by describing PC5 as a 'dehydration intermediate', which temperature-dependent population is simultaneously replenished to the expenses of fully hydrated Cu(II) complexes, and eroded in favour of framework-interacting Cu(II) sites. Interestingly, by including PC5 in our model, small populations of Z_2Cu(II) also become detectable for high-loading samples with Si/Al = 15, 19 and 29, with maximum relative abundances decreasing as the Si/Al increases (see Figure S5d, e, f).

The well-defined 'identity' of PC5, both in term of spectral shape and repeatable behaviour of the concentration profiles along the sample series, together with the R-factor analysis reported below, supports its physical-chemical meaningfulness.

The situation is more blurred in the case of PC6 found by running the MCR-ALS protocol in an upsized PC space with N_{pure} = 6. The addition of an extra-component (PC6/6 in Figure S6) causes slight re-modulations in the concentration profiles for Z[Cu(II)OH] species (PC3/6) and Cu(II) dehydration intermediates (PC5/6), whereas all the other three components are negligibly perturbed. Such behaviour is perfectly in line with the corresponding $\mu_6^{pure}(E)$, which does not show new characteristic peaks and is well approximated by an average between the $\mu_3^{pure}(E)$ and the $\mu_5^{pure}(E)$ curves. Furthermore, the concentration profiles for PC6 do not show a clear trend along the samples series, being either overlapped with the PC3 ones (Figure S6a-c) or exhibiting an intermediate peak between the PC5 and PC3 concentration maxima (Figure S6d-f).

All these observations are diagnostic for an oversized PC space, and together with the R-factor analysis reported in the following Section, discourage the possibility to reliably isolate a sixth Cu-species, at least within the spectral and temporal resolution of the dataset employed in this work.

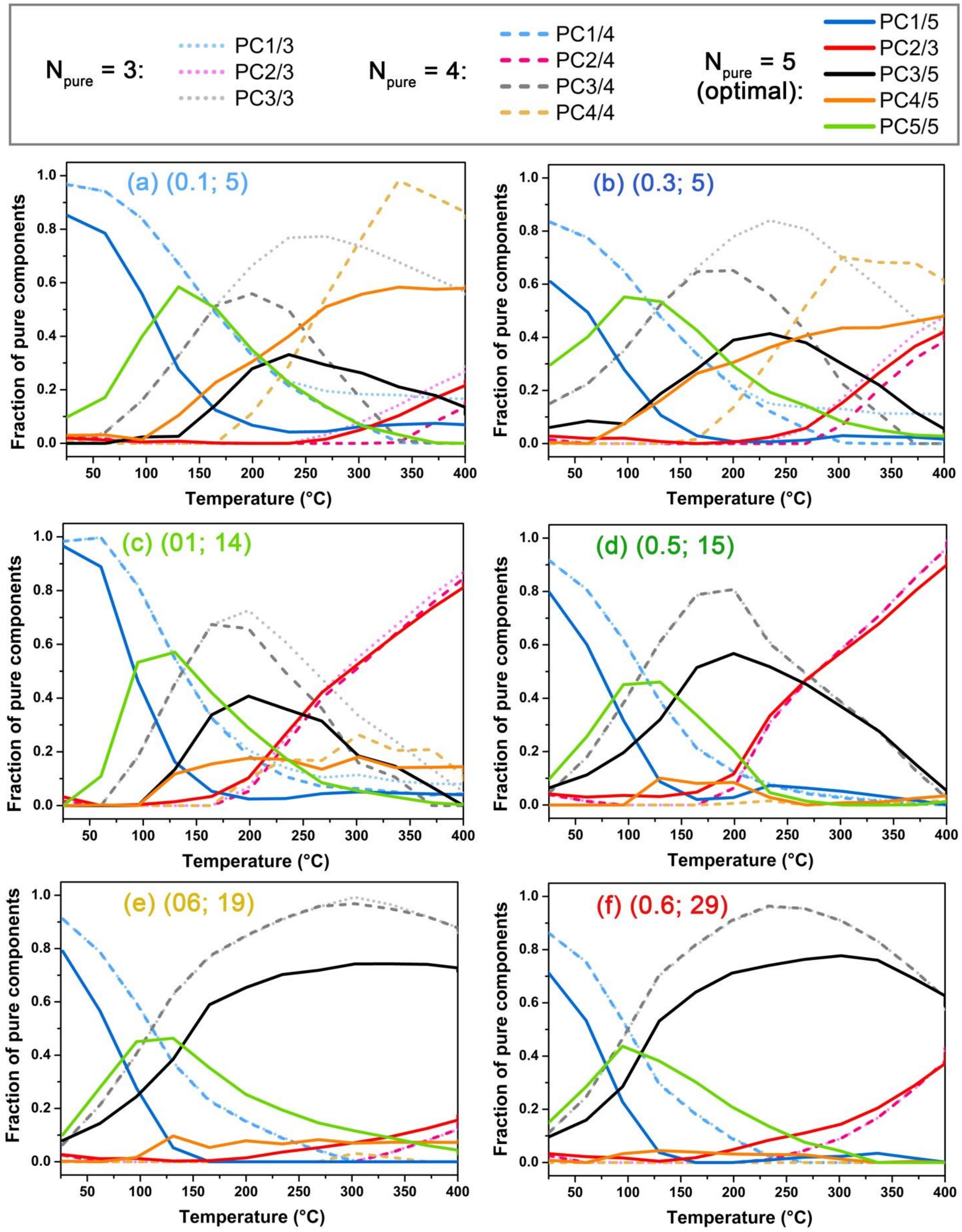

Figure S5. T-dependent concentration profiles of pure components derived from MCR-ALS using downsized (N_{pure} = 3, 4, dotted and dashed lines, respectively) and optimal (N_{pure} = 5, solid lines) PC spaces. Panels (a)-(f) report the results for each of the investigated Cu-CHA compositions, indicated with (Cu/Al; Si/Al) labels. The concentration profiles are reported using the same colour code employed in Figure S4a to show the corresponding theoretical $\mu_i^{pure}(E)$ spectra.

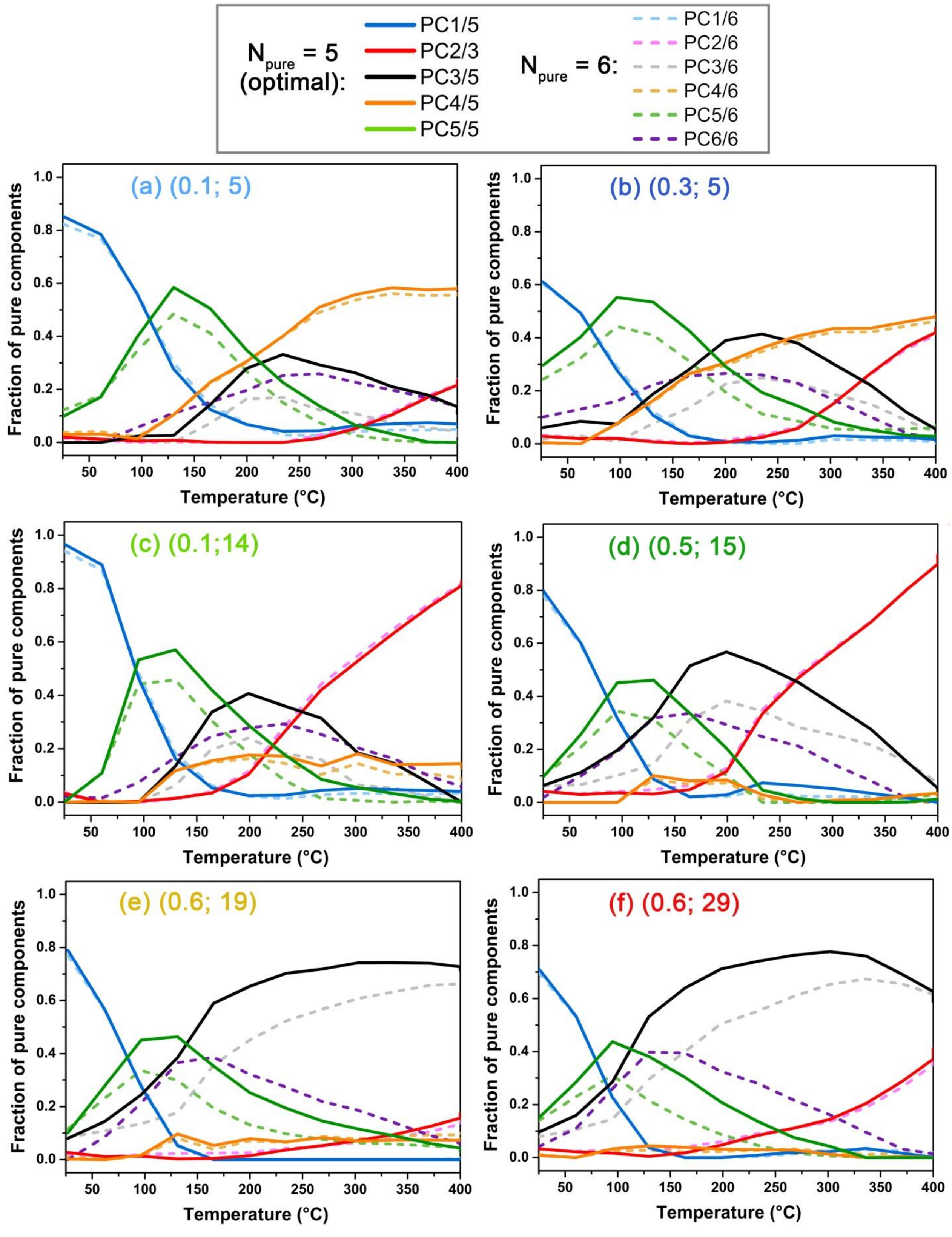

Figure S6. T-dependent concentration profiles of pure components derived from MCR-ALS using upsized (N_{pure} = 6, dashed lines) and optimal (N_{pure} = 5, solid lines) PC spaces. Panels (a)-(f) report the results for each of the investigated Cu-CHA compositions, indicated with (Cu/Al; Si/Al) labels. The concentration profiles are reported using the same colour code employed in Figure S4b to show the corresponding theoretical $\mu_i^{pure}(E)$ spectra.

3.2 R-factor analysis for MCR-ALS reconstructions in downsized or upsized PC spaces

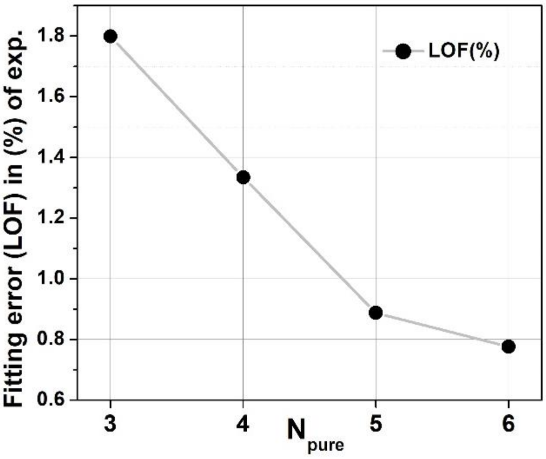

Figure S7. LOF (% exp) global quality indicator as a function of N_{pure}.

Considering a *global* quality indicator for the reconstruction such as the LOF parameter returned at the end of the MCR-ALS optimization procedure (see Section 1.4.1), the reconstruction quality monotonically increases (LOF decreases) while increasing N_{pure}, as expected. However, a saturation effect is clearly observed while moving from $N_{pure} = 5$ (identified as the optimum based on PCA and previous considerations on spectra/profiles dependency on the PC space dimension) and $N_{pure} = 6$.

To further support our assessment on the most reliable N_{pure} to select in the analysis of our dataset, herein we examine the *local* reconstruction quality as a function of the temperature and the sample composition.

To this aim, we evaluate as a function of N_{pure} the T-dependent profiles of the R-factors for each investigated composition. The R-factor parameter is commonly used to evaluate the agreement between experimental and fitted/simulated data (*e.g.* in Rietveld refinement of diffraction data or in XANES linear combination fit), and calculated as $\Sigma_j |\mu^{exp}{}_j (T, E) - \mu^{rec}{}_{,j} (T, E)| / \Sigma_j [\mu^{exp}{}_j (T, E)]$, where j indicates each energy point in the analysed energy range, (8975−9020) eV. Figure S8 reports an overview of the results, comparing the R-factor profiles obtained for the optimal $N_{pure} = 5$ value (black solid lines) with the ones obtained using downsized (dotted and dashed blue lines for $N_{pure} = 3$ and 4, respectively) or upsized (dashed-dotted red lines, $N_{pure} = 6$) PC spaces.

From Figure S8, it clearly appears how the ‘basic’ 3PC-model is not adequate to describe the high-T range in the Si/Al = 5 samples, resulting in a substantial increase of R-factor values for temperatures > 250 °C. Here, the addition of a 4th PC drastically improve the reconstruction quality, whereas for all the other compositions minor differences among 3PC- and 4PC-models are observed in term of R-factor values and temperature-dependent trends. Conversely, the transition from a 4PC- to a 5PC model has a general positive influence on the reconstruction quality for the whole sample series, albeit the most pronounced improvements are observed in different T-ranges as a function of the composition. In particular, for Si/Al = 5 samples R-factor values significantly decrease in the low-T range while for the Si/Al = 19, 20 catalysts, the most evident improvements are observed for T > 250 °C. Finally, the addition of a 6th PC generally results either in a very minor improvement or in a slight increase of R-factor values, with the only exception of the (0.1; 14) sample, where a significant improvement is observed especially for T < 200 °C.

These considerations can be summarized by considering the behaviour of the T-averaged R-factor values for the investigated compositions as a function of N_{pure}, shown in Figure S9. Whereas a progressive improvement (decrease of T-averaged R-factors) is observed while moving from $N_{pure} = 3$ to $N_{pure} = 5$, the average R-factor tend to increase again at $N_{pure} = 6$, for all samples but the (0.1; 14) one.

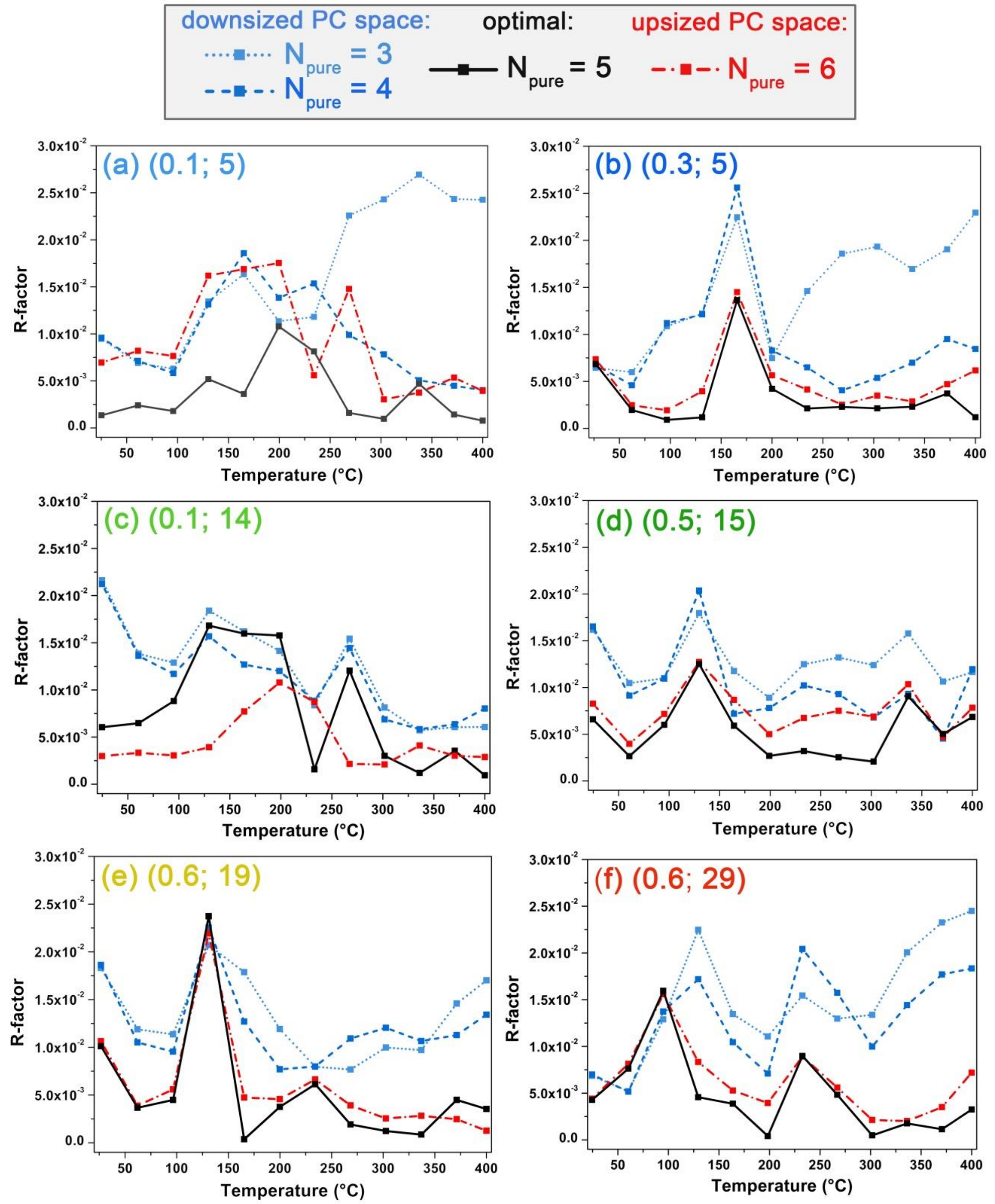

Figure S8. Comparison between the T-dependent R-factor profiles obtained for the optimal N_{pure} = 5 value (black solid lines) with the ones obtained using downsized (dotted and dashed blue lines for N_{pure} = 3 and 4, respectively) or upsized (dashed-dotted red lines, N_{pure} = 6) PC spaces. Panels (a)-(f) report the results for each of the investigated Cu-CHA compositions, indicated with (Cu/Al; Si/Al) labels.

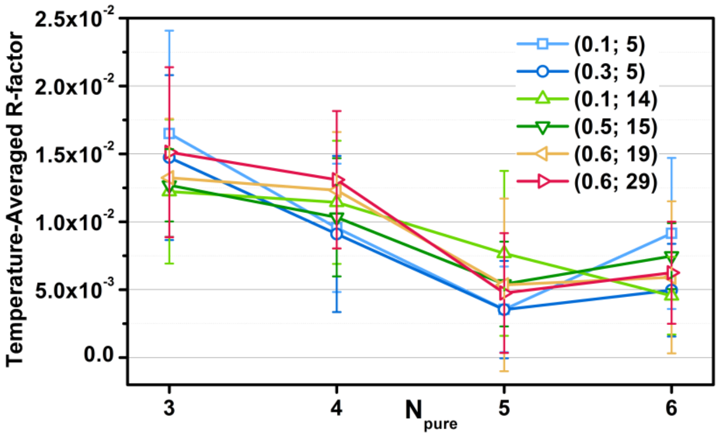

Figure S9. Behaviour of T-averaged R-factor values for the investigated Cu-CHA compositions as a function of N_{pure}. The error bars for each T-averaged R-factor correspond to the standard deviations on the average performed on the 13 available T-points in the RT−400 °C range.

According to the general trend and consistently with statistical analysis, an optimum is found for the quality of the MCR-ALS reconstruction in correspondence of $N_{pure} = 5$.

Nonetheless, it is clear that additional complexity exists in the 130−250 °C range, to be addressed into future dedicated experiments. Here, for all the probed compositions, R-factors exhibit their maxima, possibly due to composition-specific contributions from a pool of additional intermediates, which however could not be reliably assimilated in a common 6th PC. Importantly, such a temperature range demarcates the transition between homogeneous-like Cu-complexes and framework-interacting Cu species. In this range, composition-specific self-organizing phenomena could be fostered by the enhanced mobility of partially hydrated/hydroxylated Cu(II) species loosely charge-balanced by the framework anionic sites before they anchor at specific locations. With this respect, it is worth to mention the very recent study by Gao *et al.*,[31] presenting an homogeneous-phase model for low-temperature oxidation half-cycle of the SCR reaction. The proposed mechanism involves two mobile $[Cu(I)(NH_3)_2]^+$ complexes transiently forming a $[Cu(I)(NH_3)_2]^+$–O_2–$[Cu(I)(NH_3)_2]^+$ species, which then evolves to $[Cu(II)(NH_3)_2]^{2+}$–O–$[Cu(II)(NH_3)_2]^{2+}$ upon oxidation of NO to NO_2.

4 Assignment of MCR-ALS $\mu^{pure}(E)$ curves for PC1 and PC5: additional information

4.1 Comparison with experimental references

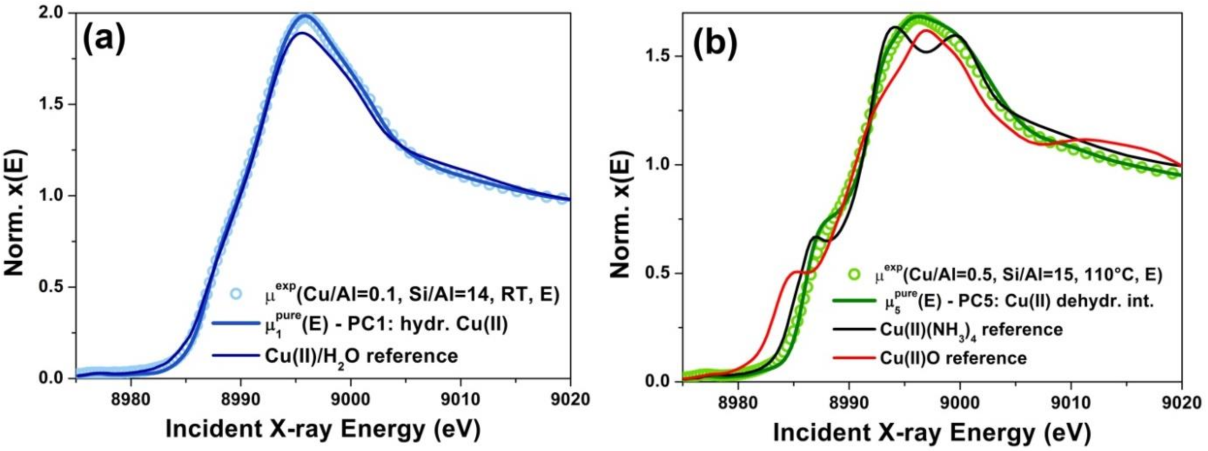

Figure S10. (a) Typical experimental XANES of Cu-CHA at RT (sample composition: Cu/Al = 0.1; Si/Al = 14) in correspondence of maximum concentration for PC1 (light blue circles) and correspondent pure theoretical XANES μ_1^{pure} (E) for PC1 (blue solid line) compared with XANES spectra of a reference aqueous solution of Cu(II) acetate (dark blue solid line). (b) Typical experimental XANES of Cu-CHA at 100 °C, sample composition: Cu/Al = 0.5; Si/Al = 15) in correspondence of maximum concentration for PC5 (green circles) and correspondent pure theoretical XANES μ_5^{pure} (E) for PC5 (solid green line) compared with XANES spectra of reference 4-fold coordinated Cu(II) species: CuO (solid red line) and $[Cu(II)(NH_3)_4]^{2+}$ (black solid line).

4.2 XANES simulations for $[Cu(II)(H_2O)_5]^{2+}$ and $[Cu(II)(H_2O)_4]^{2+}$

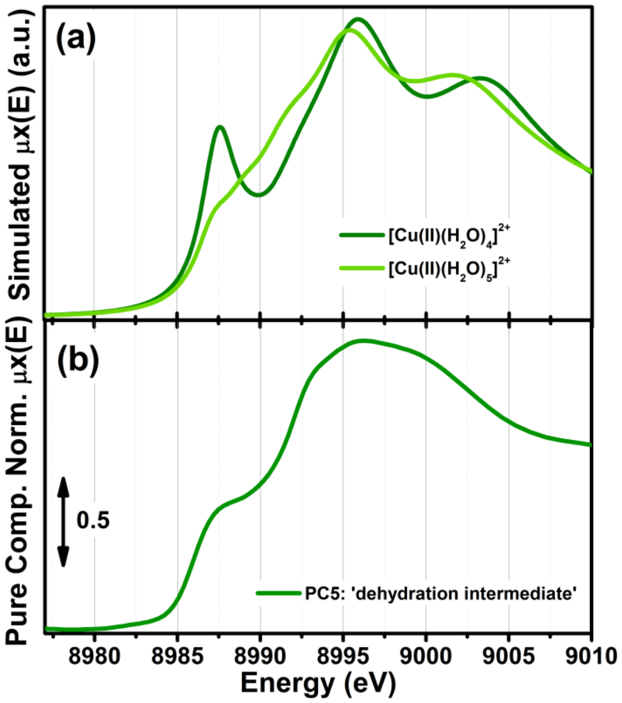

Figure S11. (a) Simulated Cu K-edge XANES spectra corresponding to different DFT-models for under-coordinated Cu(II) aquo-complexes, namely $[Cu(II)(H_2O)_5]^{2+}$ and $[Cu(II)(H_2O)_4]^{2+}$. (b) μ_5^{pure} (E) spectrum obtained from MCR-ALS analysis for PC5, assigned to a Cu(II) dehydration intermediate. From comparison between simulated and MCR-ALS spectra, it emerges that PC5 entails additional complexity, in line with the relatively higher R-factor values in the temperature range 130-250°C where its concentration is maximum (see Figure S11 and Figure 4 main text). Although the similarity with four-coordinated reference compounds (see Figure S10b) suggests predominant ligation to 4 H_2O/OH ligands, simulations evidence that μ_5^{pure} (E) spectrum could actually reflect a dynamic mix of four-, five-, and, possibly, even lower-coordinated Cu(II) aquo complexes, which are however not resolvable within time- and energy-resolution of the available dataset.

5 Details on EXAFS analysis

5.1 *EXAFS spectra in k-space*

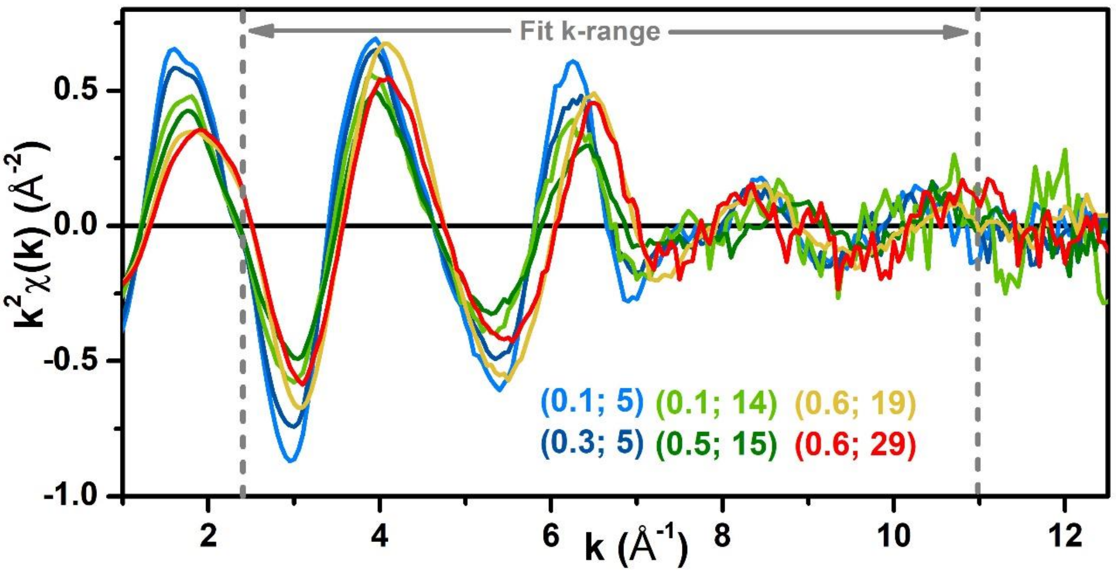

Figure S12. k^2chi(k) EXAFS spectra collected at 400 °C after He-activation on the investigated series of Cu-CHA catalysts. Sample composition is indicated using (Cu/Al; Si/Al label). The dashed grey vertical bars delimit the k-space range adopted for EXAFS fitting as described in the following sections.

5.2 *Fit details and shells model adopted for EXAFS analysis*

Both mono-component and multi-component fits of the *in situ* EXAFS spectra collected at 400 °C on the He-activated Cu-CHA catalysts were performed in R-space in the $\Delta R = 1.0 - 3.2$ Å range, on the FT of the k^2-weigthed $\chi(k)$ EXAFS spectra transformed in the 2.4 – 11.0 Å^{-1} range, resulting in 12 independent points ($2\Delta k\Delta R/\pi > 12$). Phases and amplitudes have been calculated by FEFF6 code[32, 33] using the Arthemis software from the Demeter package.[8]

As an input for the FEEF calculations, we employed DFT-optimized geometries, obtained as described in our previous study,[5] of the principal Cu-sites evidenced by XANES MCR-ALS analysis in the He-activated state, namely *1Al* Z[Cu(II)OH] in the *8r*, *1Al* ZCu(I) in both *8r* and *6r*, and *2Al* Z_2Cu(II) in the *6r*, in correspondence of both Al−Si−Al and Al−Si−Si−Al linkages. Our fitting model included all the single-scattering (SS) paths contributing in the analysed R-space range. In order to limit the number of optimized variables, we optimized all the SS paths included in the fitting model using the same passive amplitude reduction factor (S_0^2) and the same energy shift parameter (ΔE). Coordination numbers were fixed according to the relevant DFT-models (see also Section 5.3 here below).

In the employed fitting model (similar to the one used in our previous EXAFS study of Cu-CHA[5]) we identified the following coordination shells around the Cu absorber:

- First-shell extra-framework oxygen atom (**O_{ef}**), only present in the *1Al* Z[Cu(II)OH] geometry with coordination number $N_{O(ef)} = 1$; parametrized with specific radial shift $\Delta R_{O(ef)}$ and $\sigma^2_{O(ef)}$ parameters to properly account for the different chemical nature of extra-framework O-ligands with respect to the zeolite O_{fw} atoms.
- First-shell framework oxygen atoms (**O_{fw}**), present in all the examined geometries with coordination numbers $N_{O(fw)}$ in the 2−4 range; parameterized with an independent radial shift ($\Delta R_{O(fw)}$) applied to the individual DFT-optimized bond distances and Debye-Waller (DW) factor ($\sigma^2_{O(fw)}$).
- Second-shell framework oxygen atoms (**O'_{fw}**), present in the geometries for *1Al* ZCu(I) in *6r* (coordination number $N_{O'(fw)} = 2$), and *2Al* Z_2Cu(II) in *6r* with Al−Si−Al linkages (coordination number $N_{O'(fw)} = 1$; parametrized with an independent $\Delta R_{O'(fw)}$ and the same DW used for the SS contribution involving the first shell framework oxygens: $\sigma^2_{O'(fw)} = \sigma^2_{O(fw)}$.

- Second-shell framework Si/Al atoms (**T_{fw}**), present in all the geometries, with coordination number $N_{T(fw)}$ = 1 in *1Al* Z[Cu(II)OH] *8r* and *1Al* ZCu(I) *8r* and of $N_{T(fw)}$ = 2 in all the other geometries; parameterized with an independent radial shift ($\Delta R_{T(fw)}$) and DW factor ($\sigma^2_{T(fw)}$).
- SS paths involving farer Si and O neighbours of the *6r* and *8r* (**fw**), with distances from the Cu absorber in the 2.8–3.5 Å range and coordination number of N_{fw} = 6 and N_{fw} = 4 for *6r* and *8r* geometries, respectively; modelled considering a common contraction/expansion factor α_{fw} and DW factor σ^2_{fw} increasing as the square root of the distance $R_{eff,i}$ of the ith scattering atom from the absorber ($\Delta R_{fw,i} = \alpha_{fw} R_{eff,i}$, $\sigma^2_{fw,i} = ss_{fw} (R_{eff,i}/R_0)^{1/2}$, where R_0 denotes the shortest R_{eff} for the group of paths).

The number of fit parameters varied from 8 to 10, depending on the specific DFT-optimized geometries employed as starting guess, being always below the number of employed independent points ($2\Delta k\Delta R/\pi > 12$). The fit quality was evaluated considering the R-factor values (R-factor = 0: perfect agreement between experimental and theoretical curve; R-factor > 0.05: typical threshold for unsatisfactory fit) computed by the Arthemis program after each EXAFS fit.

5.3 *Results of single-component fits on the 'purest' He-activated states*

Singling out the contributions from the different Cu-sites in the EXAFS spectra collected at 400 °C after dehydration in He for the multi-composition dataset is not straightforward. Multi-component fits performed without any constrain would result in an excess of free parameters ($N_{par} >> N_{ind}$), definitely yielding not reliable results. Nonetheless, under a series of realistic hypotheses, a constrained multi-component EXAFS analysis can be carried out to obtain an independent evaluation of Cu-speciation at 400 °C to be compared with the results from MCR-ALS XANES analysis at the end of the dehydration ramp.

In particular, from MCR-ALS results (see Figure 2b in main text) we realize that the Cu-speciation at 400 °C is largely dominated by only three structural components, present in difference relative fractions at different compositions:

- *1Al* sites in their oxidized form: Z[Cu(II)OH] (assigned to PC3 in MCR-ALS analysis);
- *1Al* sites in their reduced form: ZCu(I) (assigned to PC2 in MCR-ALS analysis);
- *2Al* Z_2Cu(II) sites (assigned to PC4 in MCR-ALS analysis).

Based on these evidences, we carried out a preliminary EXAFS fitting step selecting the compositional points characterized by the 'purest' Cu-speciation at 400 °C. Each of these state is representative of major contributions from one among the three dehydrated Cu-species listed above, i.e. ca. 75% Z[Cu(II)OH] in sample (Cu/Al=0.6; Si/Al=19); ca. 90% ZCu(I) in sample (Cu/Al=0.5; Si/Al=15); ca. 60% Z_2Cu(II) in sample (Cu/Al=0.1; Si/Al=5). As starting points for the fits, we thus employed the corresponding DFT-optimized geometry/geometries, as described in the previous Section 5.2.

The results of mono-component fits on the samples showing the purest final state at 400 °C are reported in Table S4−Table S6. These fits inherently represent an approximate description of the samples, and the refined parameters will suffer of a certain level of 'contamination' from the other minor Cu-species present. However, due to the presence of a largely dominant Cu-species, the refined structural parameters represent a good starting point for a the subsequent multi-component fitting step. This is also supported by the good quality reached in the mono-component fits (at least in correspondence of the preferred structural environments, see below) in terms of R-factor and physical reliability of the optimized parameters values.

Alternative environments for Z_2Cu(II) and ZCu(I) are in principle possible. Indeed, for the Z_2Cu(II) species, sites in *6r* with either Al−Si−Al or Al−Si−Si−Al linkages could be present, whereas ZCu(I) species expected to form from reduction of Z[Cu(II)OH] complexes, could either remain in the plane of the *8r* or migrate to a neighbouring *6r*. Alternative configurations for both ZCu(I) and Z_2Cu(II) species are compared in Table S5 and Table **S6**. We have tested both the alternatives as starting guesses for mono-component fits of He-activated Cu-CHA with composition (Cu/Al=0.5; Si/Al=15) and (Cu/Al=0.1; Si/Al=5), representative of ZCu(I) and Z_2Cu(II) species, respectively, to select the best configuration among the available alternatives presented by DFT.

As shown in Table S6, for sample (Cu/Al=0.1; Si/Al=5), a test fit performed using the $Z_2Cu(II)$ Al−Si−Al configuration yielded significantly worse agreement with experimental spectrum (R-factor = 0.02826) with respect to what obtained for the Al−Si−Si−Al configuration (R-factor = 0.0065) and very high deviations from the DFT geometry (e.g. $\Delta R_{O'(fw)}$ ~ 0.2 Å). The $Z_2Cu(II)$ site hosted in *6r* with Al−Si−Si−Al linkages is hence assumed as the most likely configuration describing the PC4 species, and employed for the subsequent multi-component EXAFS analysis.

For sample (Cu/Al=0.5; Si/Al=15) the situation is less neat (see Table S5). Unfortunately, the local environment for ZCu(I) in *6r* and *8r* is difficulty distinguishable by EXAFS, albeit structurally different. Indeed, in the *6r* configuration, an intrinsic antiphase is expected between the $\mathbf{T_{fw}}$ shell ($N_{T(fw)}$ = 2, $\langle R_{T(fw)} \rangle$ = 2.81 Å from DFT) and the $\mathbf{O'_{fw}}$ shells ($N_{O'(fw)}$ = 2, $\langle R_{O'(fw)} \rangle$ = 2.51 Å from DFT), making the resulting EXAFS signal rather similar to the one deriving from ZCu(I) sites in the *8r*, where the $\mathbf{T_{fw}}$ shell has a lower coordination number $N_{T(fw)}$ = 1. Based on the much higher R-factor = 0.071 observed for ZCu(I) sites in the *8r* and, in general, on the higher quality observed using the *6r* model, we selected this latter a geometry as the most representative for ZCu(I) sites, although EXAFS analysis clearly reveals that abundant Cu(I) populations are accompanied by important levels of structural disorder (see also Section 2.4 in the main text)

Table S4. Results from mono-component EXAFS fits of He-activated Cu-CHA with composition (Cu/Al = 0.6; Si/Al = 19) using the *1Al* Z[Cu(II)OH] model. Average bond distances from Cu in the DFT-optimized geometry are reported in parentheses.

**Composition:**	**Cu-species:**
**Cu/Al = 0.6; Si/Al = 19**	**1AlCu(II)OH**
DFT-model of dominant Cu-species	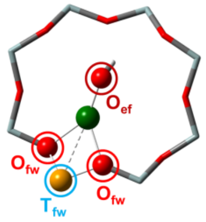
R-factor	0.0058
N_{par} (N_{ind})	8 (12)
S_0^2	1.0 ± 0.1
ΔE (eV)	− 3 ± 2
$R_{O(ef)}$ (Å)	1.81 ± 0.01 (1.76)
$\langle R_{O(fw)} \rangle$ (Å)	1.90 ± 0.04 (1.99)
$R_{T(fw)}$ (Å)	2.72 ± 0.02 (2.73)
α_{fw}	− 0.04 ± 0.01
$\sigma^2_{O(ef)}$ (Å^2)	0.007 ± 0.003
$\sigma^2_{O(fw)}$ (Å^2)	0.004 ± 0.003
$\sigma^2_{T(fw)}$ (Å^2)	0.010 ± 0.003
ss_{fw} (Å^2)	0.02 ± 0.1

Table S5. Results from mono-component EXAFS fits for He-activated Cu-CHA with composition (Cu/Al = 0.5; Si/Al = 15) using the *1Al* ZCu(I) models in both *d6r* (preferred environment) and *8r* (alternative environment). Average bond distances from Cu in the DFT-optimized geometry are reported in parentheses.

**Composition:**	**Cu-species: 1Al ZCu(I)**	
**Cu/Al = 0.5; Si/Al = 15**	**Preferred environment ZCu(I) *6r***	**Alternative environment ZCu(I) *8r***
DFT-model of dominant Cu species	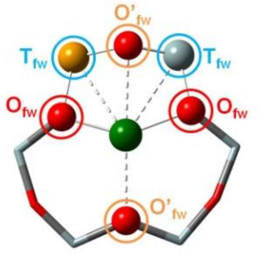	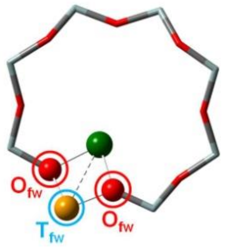
Fit R-factor	0.019	0.071
N_{par} (N_{ind})	10 (12)	8 (12)
S_0^2	1.0 ± 0.1	1.0 ± 0.1
ΔE (eV)	− 7.7 ± 0.4	− 7 ± 1
<$R_{O(fw)}$> (Å)	1.86 ± 0.02 (1.91)	1.91 ± 0.01 (1.96)
<$R_{O'(fw)}$> (Å)	2.59 ± 0.05 (2.51)	-
<$R_{T(fw)}$> (Å)	2.70 ± 0.06 (2.81)	2.73 ± 0.04 (2.71)
α_{fw}	− 0.05 ± 0.03	− 0.02 ± 0.04
$\sigma^2_{O(fw)}$ (Å^2)	0.008 ± 0.001	0.009 ± 0.001
$\sigma^2_{O'(fw)}$ (Å^2)	0.008 ± 0.001	-
$\sigma^2_{T(fw)}$ (Å^2)	0.011 ± 0.004	0.014 ± 0.005
ss_{fw} (Å^2)	0.017 ± 0.008	0.011 ± 0.008

Table S6. Results from mono-component EXAFS fits for He-activated Cu-CHA with composition (Cu/Al = 0.1; Si/Al = 5) using the *2Al* Z_2Cu(II) in 6r with either Al−Si−Al (alternative environment) or Al−Si−Si−Al linkages (preferred environment). Average bond distances from Cu in the DFT-optimized geometry are reported in parentheses.

**Composition:**	**Cu-species: *2Al* Z_2Cu(II)**	
**Cu/Al = 0.1; Si/Al = 5**	**Preferred environment Z_2Cu(II) “Al-Si-Si-Al”**	**Alternative environment Z_2Cu(II) “Al-Si-Al”**
DFT-model of dominant Cu species	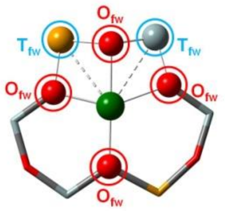	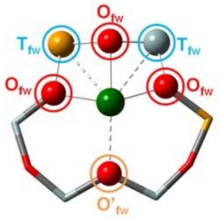
Fit R-factor	0.0065	0.017
N_{par} (N_{ind})	8 (12)	10 (12)
S_0^2	0.9 ± 0.1	0.9 ± 0.1
ΔE (eV)	− 5 ± 2	− 1 ± 2
<$R_{O(fw)}$> (Å)	1.96 ± 0.01 (2.03)	1.98 ± 0.01 (1.97)
$R_{O'}$ (Å)	-	2.62 ± 0.08 (2.39)
$R_{T(fw)}$ (Å)	2.79 ± 0.01 (2.80)	2.74 ± 0.04 (2.71)
α_{fw}	− 0.02 ± 0.01	− 0.01 ± 0.01
$\sigma^2_{O(fw)}$ (Å^2)	0.007 ± 0.002	0.003 ± 0.001
$\sigma^2_{O'(fw)}$ (Å^2)	-	0.001± 0.001
$\sigma^2_{T(fw)}$ (Å^2)	0.011 ± 0.002	0.007 ± 0.005
ss_{fw} (Å^2)	0.017 ± 0.004	0.003 ± 0.004

5.4 Details on multi-component EXAFS fits on the whole multi-composition dataset

The results from mono-component EXAFS fits have been subsequently employed as the starting point for a multi-component fitting protocol extended to the whole platform of He-activated catalysts (see Figure 4 and Table 1 in the main text).

For each sample, three sets of EXAFS paths were included in the fitting model, corresponding to each of the three Cu-sites identified in He-activated Cu-CHA, as described above. These include *1Al* Z[Cu(II)OH] in *8r*, *1Al* ZCu(I) in *6r*, and *2Al* Z_2Cu(II) in *6r* with Al−Si−Si−Al linkages. For each geometry, the structural parameters (radial shift ΔR_i for each shell of scatterers) were fixed to the best-fit values refined in correspondent mono-component fits, with a global S_0^2 set to the ideal value of 1 (also consistently with S_0^2 values found in mono-component fits, see Tables Table S4−Table S6). DW factors for the relevant shells of atomic neighbours, and, most importantly, relative fractions A_i of each component, i = Z[Cu(II)OH], ZCu(I), Z_2Cu(II), have been then guessed for each sample. The relative fractions A_i were optimized imposing $\Sigma_i A_i = 1$, i.e. that the three dehydrated Cu-species included in the fitting model are able to account for all the Cu present at 400 °C in He, within the technique sensitivity.

5.5 EXAFS fitting results in k-space

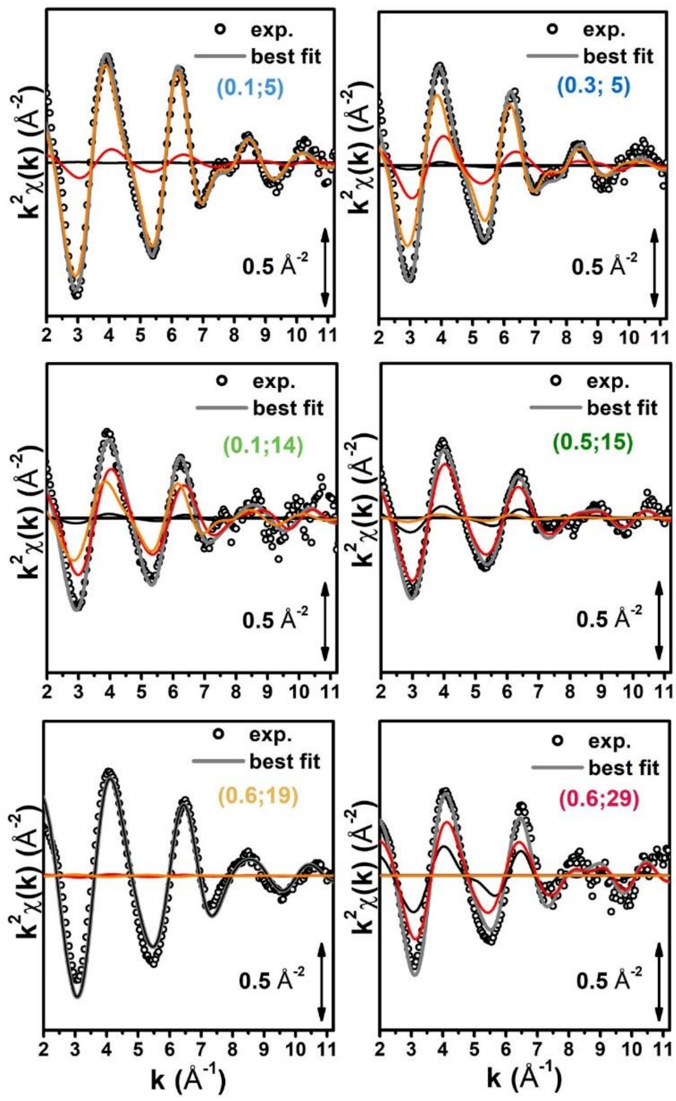

Figure S13. Comparison between experimental (black circles) and best fit (grey thick lines) $k^2\chi(k)$ EXAFS spectra obtained by multi-component fits on the whole Cu-CHA sample series after He-activation at 400 °C; for each sample, the weighted contributions from the three dehydrated Cu-species included in the fitting model are also reported as thin solid lines (*1Al* Z[Cu(II)OH]: black, *1Al* ZCu(I): red; *2Al* Z_2Cu(II): orange). Sample composition is indicated by (Cu/Al; Si/Al labels).

6 Details on IR spectral deconvolution

Deconvolution of the N_2 IR spectra reported in the main text (Figure 5a) was carried out on the high coverage curves, using the Levenberg-Marquardt algorithm with the OPUS software (Version 5.0, Bruker Optik GmbH). The curve fit analysis was first carried out on the spectrum obtained on the sample with and Cu/Al = 0.1 and Si/Al = 14. Two best fits were selected, and the corresponding details can be found to in Table S7. Even if the quality of the first fit was very good (RMS = 0.000000), a second one was attempted trying to modify peaks width in order to decrease their difference. However, a distinct difference in peaks width was obtained also in the second fit. These two set of data were used as starting point for the curve fit of the other samples. All curve fit analysis were carried out in the 2320−2270 cm^{-1} interval.

Table S7. Results of the two best fits obtained in spectral deconvolution of sample (0.1; 14), which was chosen as starting point due to best spectral definition of the two components.

FIT	Position	Intensity	Width	Integral	Shape	Residual RMS error
1	2290.78	0.024	7.44	0.193	0%Lorentz+Gauss	0.000000
	2297.34	0.038	12.51	0.508	0%Lorentz+Gauss	
2	2291.27	0.033	8.32	0.293	0%Lorentz+Gauss	0.000639
	2298.53	0.033	11.26	0.407	0%Lorentz+Gauss	

In the subsequent curve fit analysis, the components detailed in Table S7 were used as an input. Several fits were made, separately fixing peak widths and shape (% of Lorentzian and Gaussian contribution), assuming as a first approximation that these parameters should be intrinsic of the investigated species. Results were discarded, irrespective of the RMS error, when not physically meaningful, particularly when the position of the resulting peaks was too far from the expected/average values. All acceptable results and corresponding residual RMS errors are listed in Table S8-Table S10. Analysis was carried out on the spectra before normalization for pellet thickness and Cu loading (see Section 1.3).

For both samples (0.5; 15) and (0.6; 29) no acceptable fits could be obtained by fixing peak widths as in FIT1 and 2 of Table S7. Additional curve fit analysis were carried out without any constraints, starting from inputs from Table S7 or fixing the two initial components manually. The accepted fits (Table S8Table S9) gave peak widths and positions in good agreement with respect to the values obtained on sample (0.1; 14).

In the case of sample (0.1; 5) good results were obtained fixing peak widths as in FIT2 of Table S7. In the other attempts, the inputs from Table S7 were used as starting point, without any constraints. In this case acceptable fits were obtained, even if with a considerable broader LF peak with respect to the average of the analysed catalysts. In this case, curve fit was also carried out using a single HF component (FIT6 in Table S10).

Table S8. Results of the curve fit analysis carried out on sample (0.5;15). Stars indicate fixed parameters.

FIT	Position	Intensity	Width	Integral	Shape	Residual RMS error:
1	2292.31	0.339	5.07	1.830	0%Lorentz+Gauss *	0.001957
	2296.16	0.267	12.29	3.499	0%Lorentz+Gauss *	
2	2292.29	0.366	5.23	2.171	14%Lorentz+Gauss	0.00114
	2296.5	0.253	11.81	3.277	6%Lorentz+Gauss	
3	2292.29	0.344	5.091	1.866	0%Lorentz+Gauss *	0.001467
	2296.20	0.266	12.09	3.435	0%Lorentz+Gauss *	
4	2292.2	0.103	5.14	0.577	5%Lorentz+Gauss	0.000421
	2296.29	0.076	11.99	0.974	0%Lorentz+Gauss	
5	2292.29	0.100	5.093	0.545	0%Lorentz+Gauss	0.000427
	2296.20	0.078	12.076	1.003	1%Lorentz+Gauss	

Table S9. Results of the curve fit analysis carried out on sample (0.6; 29). Stars indicate fixed parameters.

FIT	Position	Intensity	Width	Integral	Shape	Residual RMS error:
1	2291.74	0.262	6.77	1.890	0%Lorentz+Gauss *	0.001754
	2297.66	0.070	9.71	0.730	0%Lorentz+Gauss *	
2	2291.69	0.205	6.19	1.354	0%Lorentz+Gauss *	0.001676
	2295.03	0.010	12.84	1.367	0%Lorentz+Gauss *	
3	2291.23	0.075	6.80	0.540	0%Lorentz+Gauss	0.000858
	2295.82	0.023	14.33	0.393	26%Lorentz+Gauss	
4	2291.33	0.085	7.151	0.646	0%Lorentz+Gauss	0.000802
	2298.32	0.019	10.21	0.309	100%Lorentz+Gauss	

Table S10. Results of the curve fit analysis carried out on sample (0.1; 5). Stars indicate fixed parameters.

FIT	Position	Intensity	Width	Integral	Shape	Residual RMS error:
1	2292.83	0.005	12.56	0.101	86%Lorentz+Gauss	0.000066
	2299.20	0.022	10.89	0.261	0%Lorentz+Gauss	
2	2290.71	0.003	8.31 *	0.045	100%Lorentz+Gauss	0.000259
	2298.87	0.025	11.26 *	0.309	9%Lorentz+Gauss	
3	2289.46	0.003	8.31 *	0.030	0%Lorentz+Gauss *	0.000376
	2298.84	0.025	11.26 *	0.305	0%Lorentz+Gauss *	
4	2290.078	0.006	13.12	0.083	0%Lorentz+Gauss	0.000333
	2298.79	0.052	11.33	0.625	0%Lorentz+Gauss	
5	2292.66	0.008	14.75	0.137	0%Lorentz+Gauss	0.000307
	2298.98	0.048	11.12	0.571	0%Lorentz+Gauss	
6	2298.30	0.054	11.92	0.751	21%Lorentz+Gauss	0.00147

The average values of normalized integrated areas of HF and LF components for each samples are plotted in Figure S14, including standard deviations calculated from Table S7-S10. Figure S15 reports an example of the curves obtained by fit analysis.

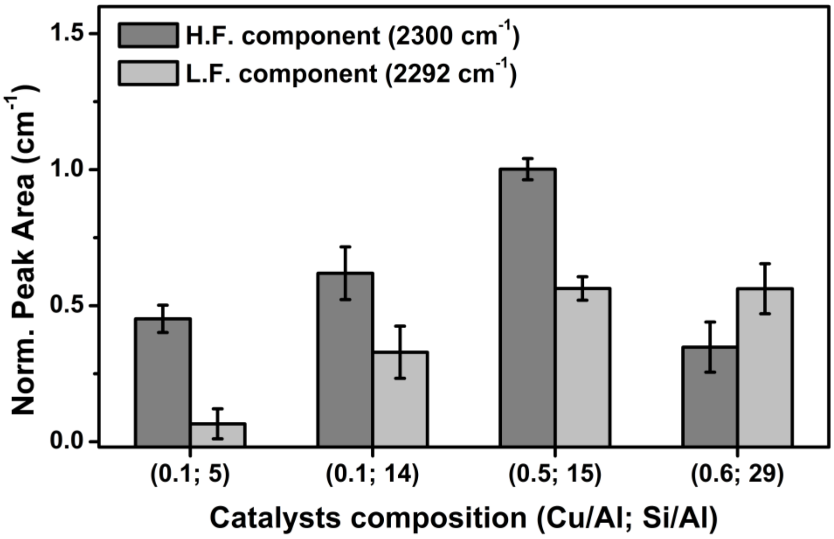

Figure S14. Normalized integrated average peak areas of HF and LF components for each of the studied samples. Average and standard deviation (error bars) were calculated after normalizing the integrated areas reported in Table S7-S10 for pellet thickness and Cu loading.

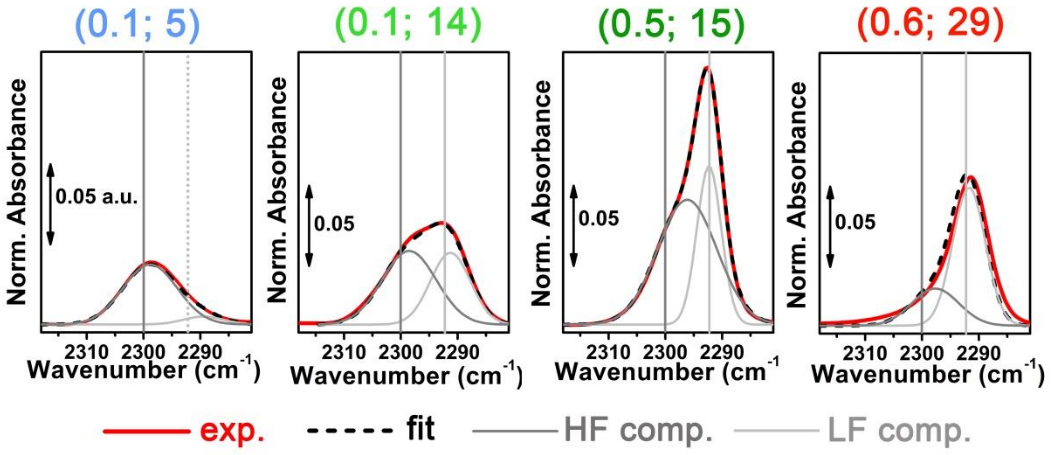

Figure S15. Experimental low-temperature normalized IR spectra corresponding to maximum coverage of N_2 adsorbed on vacuum-activated Cu-CHA catalysts, compared to typical fit curves (black dashed lines) obtained as described above. Deconvoluted HF and LF components are also reported for each fit example, as grey and light grey thin lines, respectively.

7 References

1. T. V. W. Janssens, H. Falsig, L. F. Lundegaard, P. N. R. Vennestrøm, S. B. Rasmussen, P. G. Moses, F. Giordanino, E. Borfecchia, K. A. Lomachenko, C. Lamberti, S. Bordiga, A. Godiksen, S. Mossin and P. Beato, *ACS Catal.*, 2015, **5**, 2832-2845.
2. T. D. Pham, Q. L. Liu and R. F. Lobo, *Langmuir*, 2013, **29**, 832-839.
3. D. Bellet, B. Gorges, A. Dallery, P. Bernard, E. Pereiro and J. Baruchel, *J. Appl. Crystallogr.*, 2003, **36**, 366-367.
4. F. Giordanino, E. Borfecchia, K. A. Lomachenko, A. Lazzarini, G. Agostini, E. Gallo, A. V. Soldatov, P. Beato, S. Bordiga and C. Lamberti, *J. Phys. Chem. Lett.*, 2014, **5**, 1552-1559.
5. E. Borfecchia, K. A. Lomachenko, F. Giordanino, H. Falsig, P. Beato, A. V. Soldatov, S. Bordiga and C. Lamberti, *Chem. Sci.*, 2015, **6**, 548-563.
6. S. Bordiga, E. Groppo, G. Agostini, J. A. van Bokhoven and C. Lamberti, *Chem. Rev.*, 2013, **113**, 1736-1850.
7. C. Lamberti, S. Bordiga, F. Bonino, C. Prestipino, G. Berlier, L. Capello, F. D'Acapito, F. Xamena and A. Zecchina, *Phys. Chem. Chem. Phys.*, 2003, **5**, 4502-4509.
8. B. Ravel and M. Newville, *J. Synchrotron Radiat.*, 2005, **12**, 537-541.
9. C. Ruckebusch, *Resolving Spectral Mixtures: With Applications from Ultrafast Time-Resolved Spectroscopy to Super-Resolution Imaging*, Elsevier Science, 2016.
10. E. R. Malinowski, *Factor analysis in chemistry*, John Wiley & Sons, New York, 2002.
11. W. Windig and J. Guilment, *Anal. Chem.*, 1991, **63**, 1425-1432.
12. J. Jaumot, R. Gargallo, A. de Juan and R. Tauler, *Chemometrics Intell. Lab. Syst.*, 2005, **76**, 101-110.
13. ADF2012, SCM, Theoretical Chemistry, Vrije Universiteit, Amsterdam, The Netherlands, http://www.scm.com).
14. G. te Velde, F. M. Bickelhaupt, E. J. Baerends, C. F. Guerra, S. J. A. Van Gisbergen, J. G. Snijders and T. Ziegler, *J. Comput. Chem.*, 2001, **22**, 931-967.
15. E. van Lenthe and E. J. Baerends, *J. Comput. Chem.*, 2003, **24**, 1142-1156.
16. J. P. Perdew, K. Burke and M. Ernzerhof, *Phys. Rev. Lett.*, 1996, **77**, 3865-3868.
17. E. van Lenthe, A. Ehlers and E. J. Baerends, *J. Chem. Phys.*, 1999, **110**, 8943-8953.
18. G. Smolentsev, A. V. Soldatov, J. Messinger, K. Merz, T. Weyhermuller, U. Bergmann, Y. Pushkar, J. Yano, V. K. Yachandra and P. Glatzel, *J. Am. Chem. Soc.*, 2009, **131**, 13161-13167.
19. M. Fernandez-Garcia, C. M. Alvarez and G. L. Haller, *J. Phys. Chem.*, 1995, **99**, 12565-12569.
20. J. J. Rehr and A. L. Ankudinov, *Coord. Chem. Rev.*, 2005, **249**, 131-140.
21. J. J. Kas, K. Jorissen and J. J. Rehr, in *X-Ray Absorption and X-ray Emission Spectroscopy: Theory and Application*, eds. J. A. van Bokhoven and C. Lamberti, John Wiley & Sons, Chichester (UK), 2016, pp. 73-98.
22. C. Lamberti, S. Bordiga, M. Salvalaggio, G. Spoto, A. Zecchina, F. Geobaldo, G. Vlaic and M. Bellatreccia, *J. Phys. Chem. B*, 1997, **101**, 344-360.
23. A. Iglesias-Juez, A. Martinez-Arias, A. B. Hungria, J. A. Anderson, J. C. Conesa, J. Soria and M. Fernandez-Garcia, *Appl. Catal. A-Gen.*, 2004, **259**, 207-220.
24. S. J. A. Figueroa and S. J. Stewart, *J. Synchrot. Radiat.*, 2009, **16**, 63-68.
25. H. W. P. Carvalho, S. H. Pulcinelli, C. V. Santilli, F. Leroux, F. Meneau and V. Briois, *Chem. Mat.*, 2013, **25**, 2855-2867.

26. W. H. Cassinelli, L. Martins, A. R. Passos, S. H. Pulcinelli, C. V. Santilli, A. Rochet and V. Briois, *Catal. Today*, 2014, **229**, 114-122.
27. C. Brouder and J. Goulon, *Physica B*, 1989, **158**, 351-354.
28. T. Fujikawa, *J. Phys. Soc. Jpn.*, 1999, **68**, 2444-2456.
29. A. V. Poiarkova and J. J. Rehr, *Phys. Rev. B*, 1999, **59**, 948-957.
30. H. Arai, N. Ueno and T. Fujikawa, in *X-Ray Absorption Fine Structure-Xafs13*, eds. B. Hedman and P. Painetta, Amer Inst Physics, Melville, 2007, vol. 882, pp. 108-110.
31. F. Gao, D. Mei, Y. Wang, J. Szanyi and C. H. F. Peden, *J. Am. Chem. Soc.*, 2017, **139**, 4935–4942.
32. S. I. Zabinsky, J. J. Rehr, A. Ankudinov, R. C. Albers and M. J. Eller, *Phys. Rev. B*, 1995, **52**, 2995-3009.
33. J. J. Rehr and R. C. Albers, *Rev. Mod. Phys.*, 2000, **72**, 621-654.
